# The structure of the human ABC transporter ABCG2 reveals a novel mechanism for drug extrusion

**DOI:** 10.1038/s41598-017-11794-w

**Published:** 2017-10-23

**Authors:** Narakorn Khunweeraphong, Thomas Stockner, Karl Kuchler

**Affiliations:** 1 Center for Medical Biochemistry, Max F. Perutz Laboratories, Medical University of Vienna, Campus Vienna Biocenter, Dr. Bohr-Gasse 9/2, A-1030 Vienna, Austria; 2 Center for Physiology and Pharmacology, Institute of Pharmacology, Medical University Vienna, Währingerstrasse 13A, A-1090 Vienna, Austria

## Abstract

The human ABC transporter ABCG2 (Breast Cancer Resistance Protein, BCRP) is implicated in anticancer resistance, in detoxification across barriers and linked to gout. Here, we generate a novel atomic model of ABCG2 using the crystal structure of ABCG5/G8. Extensive mutagenesis verifies the structure, disclosing hitherto unrecognized essential residues and domains in the homodimeric ABCG2 transporter. The elbow helix, the first intracellular loop (ICL1) and the nucleotide-binding domain (NBD) constitute pivotal elements of the architecture building the transmission interface that borders a central cavity which acts as a drug trap. The transmission interface is stabilized by salt-bridge interactions between the elbow helix and ICL1, as well as within ICL1, which is essential to control the conformational switch of ABCG2 to the outward-open drug-releasing conformation. Importantly, we propose that ICL1 operates like a molecular spring that holds the NBD dimer close to the membrane, thereby enabling efficient coupling of ATP hydrolysis during the catalytic cycle. These novel mechanistic data open new opportunities to therapeutically target ABCG2 in the context of related diseases.

## Introduction

ABC transporters comprise one of the largest families of ubiquitous membrane transport proteins in all kingdoms of life operating as exporters and importers for a remarkable range of substrates^1^. Moreover, inborn errors in human ABC genes cause prominent genetic diseases, including cystic fibrosis^2^, gout^3^, as well as lipid disorders^4^. Importantly, many ABC transporters are responsible for, or associated with, resistance to anticancer and anti-infective drugs and thus of considerable medical relevance^5^.

The minimal functional unit of a membrane-embedded ABC transporter has two highly conserved nucleotide binding domains (NBDs) connected to two integral transmembrane domains (TMDs), each usually containing 6–10 putative transmembrane-spanning helices (TMHs). The dimeric NBDs energize the transport cycle via ATP hydrolysis using a mechanism^6,7^ that remained obscure or at least a matter of controversy^8–12^. While the alternating access model may apply for both ABC importers^13^ and exporters^10,14,15^, accumulating evidence challenges previous notions of a unifying transport mechanism for all ABC transporters^9,11^. Thus, despite high structural conservation, distinct molecular mechanisms and transport cycles are likely to exist^16,17^.

The human ABCome contains 48 genes encoding ABC proteins classified into 7 subfamilies from ABCA to ABCG^18^. The breast cancer resistance protein, BCRP or ABCG2 is a prototypic multidrug resistance transporter engaging in a ménage a trois with P-gp (ABCB1) and MRP1 (ABCC1) in clinical anticancer resistance^19^, though the extent of their involvement has remained a matter of discussion^20^. The detrimental roles of ABC resistance transporters are balanced by their pivotal roles in physiological detoxification^21^, where ABCG2, MRP and P-gp act as brothers in arms in the hepatobiliary system, and in most epithelial/endothelial barriers^22–24^. Of note, ABCGs are the closest orthologues of yeast pleiotropic drug resistance (PDR) pumps implicated in clinical antifungal drug resistance^25^, including Pdr5, the best-characterized fungal drug efflux transporter^26–28^.

Recent exciting near-atomic structures of the cystic fibrosis transmembrane conductance regulator CFTR^29,30^, the SUR1 sulphonyl urea receptor^31,32^, P-gp from various species^33^, mitochondrial ABCB10^34^, a bacterial TAP orthologue^35^, and human MRP1^36^ represent significant progress towards obtaining new mechanistic information. Surprisingly, however, despite radically different physiological functions of MRP1, P-gp, CFTR and SUR, they seem to adopt similar folds^37^. By sharp contrast, the atomic structure of the ABCG5/G8 sterol transporter shows that the ABCG subfamily adopts a novel fold that is unique in the ABC family^38^. Hence, distinct transport mechanisms for ABCG2 and other ABCG family members seem plausible, although more questions than answers persist about the catalytic cycles^39^.

Hence, using the recently reported x-ray crystal structure of ABCG5/G8, we generated a robust and testable atomic model of ABCG2. Importantly, we employ extensive molecular-genetic analysis to validate structural as well as mechanistic predictions of the model. Here, we show that the charged residues residing in the NBD dimer, in the predicted first intracellular loop (ICL1) and in the elbow helix contribute to a dynamic architecture of the transmission interface that surrounds a central substrate binding cavity. The transmission interface is essential for ABCG2 folding, surface targeting, transport function, substrate recognition, as well as ATP consumption during the catalytic cycle. The glutamic acid residue E451 marking the cytoplasmic start of ICL1 is absolutely essential for function. Interestingly, salt bridge interactions of E451 with K473 and E458 with R383 in the elbow helix stabilize the transmission interface. Remarkably, E451D mutants display hyperactivated ATPase activities but remain transport-incompetent, suggesting that drug recognition and ATP hydrolysis are not necessarily coupled. The structure shows that a flexible ICL1 secures the NBD in close proximity to the cytoplasmic lipid bilayer. The transmission interface may act as a molecular clutch to promote the conformational switch preceding substrate extrusion through the outward-open state. Based on these data, we propose a new mechanism for ABCG2-mediated drug transport, whereby the dynamics of the transmission interface represents a vital part of the catalytic cycle such that it couples ATP consumption at the NBDs with drug efflux. Our data identify novel ABCG2 structural domains and highlight essential residues within the structure that are amenable to therapeutic modulation in case of ABCG2-related diseases such as anticancer resistance or gout.

## Results

### The atomic model of ABCG2 predicts distinct structural domains

We carried out a comprehensive sequence analysis of ABCG-related pumps, including relevant fungal PDR transporters (Fig. S1). ABCG5 and ABCG8 share about 41–44% similarity and 25% sequence identity with ABCG2. Moreover, the prediction of transmembrane-spanning helices (TMHs) and secondary structure (Tables S1, S2) suggested that the shared NBD-TMD configuration of mammalian ABCGs and yeast PDRs (Fig. S1B) adopt similar folds and preserve essential structural elements. As for ABCG5/G8, on top of six putative TMHs in ABCG2, we identified two additional helical stretches, with the first one directly preceding TMH1 (elbow or connecting helix), and the second (re-entry helix) immediately following TMH5 (Fig. S2A,B).

**Figure 1:**
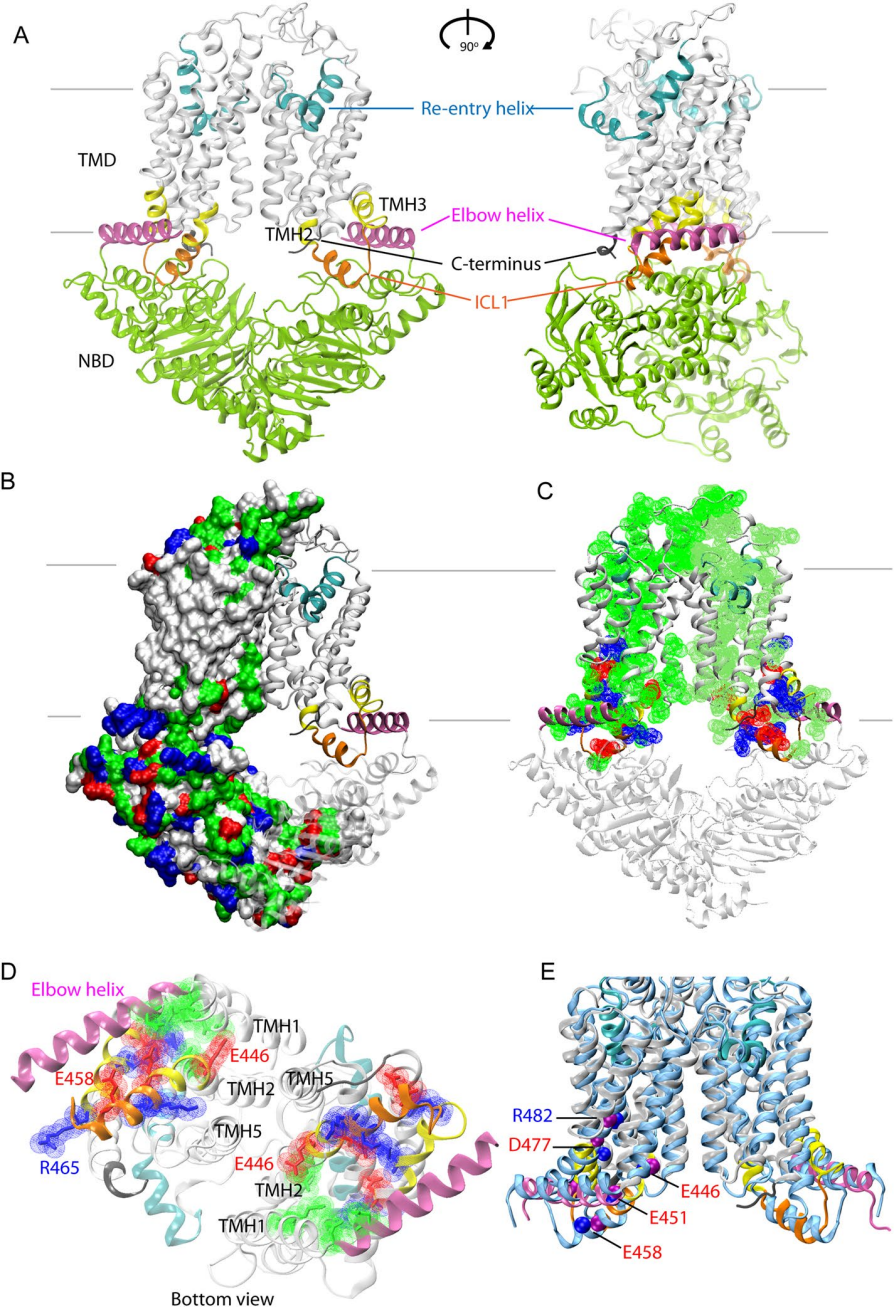
The structural model of ABCG2 reveals a novel configuration of the transmission interface. (**A**) Model of the homodimeric ABCG2 transporter in ATP-free state based on coordinates of ABCG5/G8 PDB ID: 5D07 in two views rotated by 90°. The conformation shows pronounced symmetry, including three distinct structural features forming the transmission interface in each monomer, including the elbow helix (pink), the first intracellular loop ICL1 (orange), and the surface of the NBD dimer (green); the extracellular re-entry helix (cyan) is protruding into the outer leaflet; cytoplasmic distal helical parts of TMH2 and TMH3 (yellow); C-terminus (black); TMHs (light grey), TMD, transmembrane domain, NBD, nucleotide binding domain. (**B**) Polar residues on the ABCG2 surface. The ABCG2 monomer based on ABCG5 shows residues as surface potential filling; positive charges (blue), negative charges (red), polar (green), non-polar (white). The second half is based on ABCG8 using the same color-coding of domains as above. (**C**) Polar residues in the TMD of ABCG2 molecule. (**D**) Inside-outside bottom view of ABCG2 TMDs with the NBDs removed for better overview. (**E**) Superimposition of TMDs in the cryo-EM structure^40^ of ABCG2 (PDB ID: 5Nj3) (blue) and the atomic model obtained in this study using a ribbon representation limited to the TMD regions. Color code as in panel A. Blue balls indicate residue positions, whereas purple balls indicate residues in the present atomic model. The ABCG2 cryo-EM structure PDB ID: 5Nj3 corresponds to a ABCG5 homodimer, while the present atomic model is based on the ABCG5/G8 heterodimer ﻿PDB ID: 5D07.

We then used the crystal structure coordinates of ABCG5/G8 (PDB ID: 5DO7)^38^ to create an atomic model of ABCG2 (Fig. 1). The model shows a compact homodimeric fold, in which both NBDs are in close proximity to the TMDs, with the presence of the central cavity in the transporter core. The elbow helix is amphipathic (F373 to G390) and oriented in parallel to the inner membrane leaflet. The second predicted helical region is the kinked re-entry helix (T560 to G588 containing proline in the middle) forming part of the putative third extracellular loop (ECL3). Both helical stretches are anchored in the inner and outer leaflet of the membrane bilayer, respectively (Fig. S3). Of note, ICL1 holds short helical stretches and connects TMH2 to TMH3 between positions E451 and R465. Thus, the model predicts ICL1 to be shorter than suggested by membrane topology predictions (Figs 1 and S3).

The distribution of polar residues shows that the surface of both TMDs is covered with uncharged residues that facilitate embedding in the lipid membrane (Fig. 1B). Interestingly, the core of each TMD is strikingly different, since it is largely hydrophilic and contains several charged and polar residues. Polarity extends from the elbow helix through the TMHs to reach the hydrophilic ECLs, thereby forming a roof-like polar structure that stretches across the entire membrane bilayer (Fig. 1C). The compact hydrophilic inner core of TMDs includes a polar relay^38^, which is also seen from the outside-inside view (Fig. 1D). In addition, the ABCG2 transmission interface entails a distinct structural arrangement composed of three main features, which are i) the upper part of the NBD, ii) the elbow helix, and iii) ICL1 including the short proximal helical stretches which are part of TMH2 and TMH3 (Figs 1A and S3). Strikingly, a new cryo-EM structure of ABCG2 was just published while this manuscript was in submission stage^40^. The cryo-EM structure of ABCG2 (PDB ID: 5Nj3) was obtained independently from this work and essentially corresponds to the atomic model presented here, as well as to another ABCG2 homology model recently reported^41^. In-depth comparison of the present atomic model with the cryo-EM structure PBD ID: 5Nj3, revealed almost superimposable structures (Fig. 1E). The arrangement and architecture of the transmission interface including their key residues, especially in the ABCG5 half, which is more closely related to ABCG2 than ABCG8, are at identical positions or extremely close to each other (Figs 1E and S1).

### Charged residues in the transmission interface are critical for ABCG2 function

**Figure 2:**
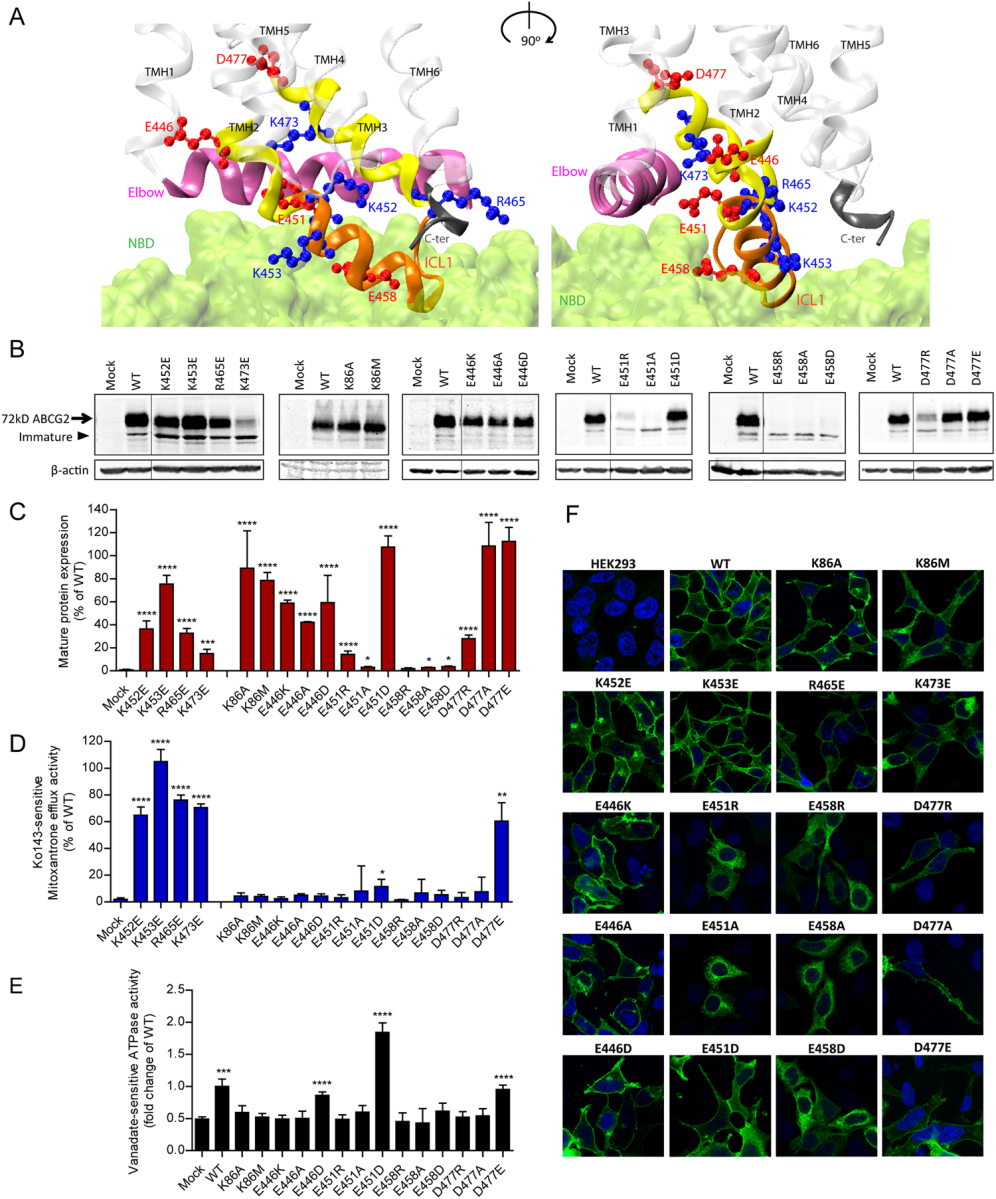
Mutational analysis of the transmission interface. All charged resideus in the predicted TMH2, ICL1 and TMH3 were subjected to mutational changes as indicated. (**A**) Configuration at the transmission interface of a human ABCG2 monomeric half transporter in two views rotated by 90°, emphasizing the contacts of the transmembrane domain (TMD) with the NBD surface (green); ICL1 (orange); TMHs (transparent white); distal helical parts of TMH2 and TMH3 (yellow); elbow helix (pink); C-terminus (black). Charged residues are indicated as colored balls-and-sticks, positive charges (blue), negative charges (red); not drawn to atomic scale. (**B**) Immunodetection of ABCG2 variants using the monoclonal mouse anti-ABCG2 (BXP-21) antibody. Mature wild type (WT) ABCG2 migrates to approximately 72 kD, the bands below are immature unglycosylated monomers. β-actin was used as a loading control. (**C**) Quantified immunoblot using the Odyssey system from several independent experiments (n = 2–12). Mature ABCG2 and β-actin were gated, and ABCG2 signals were individually normalized to β-actin and shown as a percentage of WT. The blots from different gels were divided by the white spaces. (**D**) Mitoxantrone efflux in transfected HEK293 cells after incubation at 37 °C for 20 min in the presence and absence of Ko143. Ko143–sensitive mitoxantrone efflux is given as percentage relative to WT. Data are from several independent experiments (n = 2–7). (**E**) Vanadate-sensitive ATPase activity of ABCG2 variants in transfected HEK293 cells. Vanadate-sensitive ATPase activity is represented as a fold change relative to WT. Data are from several independent experiments (n = 8) using three batches of membrane preparations. The ATPase-dead mutants, K86A and K86M served as additional controls. All data are shown as mean and SEM. ****P < 0.0001; ***P < 0.001; **P < 0.01; *P < 0.1 vs. empty plasmid transfected HEK293 (mock). (**F**) Membrane localizations of GFP-tagged ABCG2 variants were visualized by confocal microscopy to detect GFP-tagged ABCG2 variants (green). Nuclear DNA was stained with DAPI (blue). Microscopy data are from duplicate experiments.

The coupling of ATP hydrolysis to a conformational switch triggering drug transport is facilitated by the so-called coupling helix^6,9,42,43^. However, the transmission interface architecture in ABCG2 implies a novel transport mechanism. The ABCG2 model indicates that ICL1 may act as a coupling device, since it is a key part of the transmission interface, sharing surfaces with the elbow helix as well as reaching into the NBD surface (Fig. 2A). A total of eight charged residues (E446, E451, K452, K453, E458, R465, K473 and D477) are present in the region from distal TMH2, ICL1 and distal TMH3. In addition, three lysines (K647, K652, K653) are at the C-terminus after TMH6 (Fig. S2B). To test their functional role, we subjected ABCG2 to mutational analysis by introducing charge reversal mutations (i.e. K/R to D/E), aliphatic substitutions, including N-terminal tagged GFP-ABCG2 variants of all mutants. Relevant ABCG2 variants were stably expressed in HEK293 cells and subjected to functional testing, including expression level, drug efflux, ATPase activity, protein stability (Fig. S4) and targeting (Fig. S5). The functionality of GFP-ABCG2 variants were verified using mitoxantrone efflux assays (Fig. S6). All ABCG2 protein expression data of mutant variants were representedrelative to WT ABCG2.

While the positively charged residues K453, R465, K647, K652 and K653 are not conserved in the ABCG family (Fig. S1B), K473 is highly conserved. Charge-reversal mutations, including K452E, K453E, R465E were found at diminished levels on the membrane but showed only mildly altered mitoxantrone efflux (Fig. 2B–F). Likewise, C-terminal changes such as K647E, K652E and K653E significantly reduced mature levels (Fig. S7D,E), yielding distinct patterns of immature ABCG2 bands (Fig. S7D), while maintaining almost normal efflux function similar to K473E.

Remarkably, and by sharp contrast, mutational changes of all negative residues such as E466K, E451R, E458R and D477R destroyed mitoxantrone efflux (Fig. 2D). Hence, we further scrutinized the stretch from E446 to D477 (Fig. S3) by introducing alanine and glutamate or aspartate. E446 in TMH2 orients its side chain into the central cavity. The ABCG2 variants E446K/A/D only mildly impaired expression levels or targeting, but completely disrupted mitoxantrone transport (Fig. 2B–D,F). Thus, E446 is indispensable for function, though it is not conserved in the ABCG family (Fig. S1B). To check whether the loss of function was due to defective ATP hydrolysis, we quantified the vanadate-sensitive ATPase activities of all ABCG2 variants, including the kinase-dead K86A and K86M Walker A mutant controls^44,45^. Both K86A and K86M were expressed at WT levels and properly membrane-localized (Fig. 2B–D,F), but incapable of hydrolyzing ATP (Fig. 2E). WT ABCG2 displayed a 2-fold higher ATPase activity than the mock control, whereas E446K and E446A lacked ATPase activity. Most strikingly, and unexpectedly, however, the transport-incompetent E446D variant maintained ATPase activity at almost WT levels (Fig. 2E), demonstrating that E446 is important for both ATPase activity and drug efflux.

E451 marks the exit from TMH2 into ICL1 (Fig. 2A). Notably, E451 is among the most conserved residues in mammalian ABCGs (Fig. S1B), implying a critical role for function. Indeed, E451A and E451R mutations completely abolished protein levels, mitoxantrone efflux, as well as ATP hydrolysis (Fig. 2B–F). Interestingly, E451D harboring the conservative change to aspartate still lacked mitoxantrone efflux but restored mature protein levels and surface sorting. Strikingly though, E451D showed a strongly enhanced constitutive ATPase activity at about 2-fold higher levels than WT (Fig. 2E). Taken together, these data suggest that E451 at the membrane-cytoplasmic border of TMH2 is absolutely essential for ABCG2 function, and show that the negative charge must come from a glutamate.

Residue E458 is also extremely conserved among all mammalian ABCGs implying functional relevance (Fig. S1B). It locates to the bottom of ICL1 touching the NBD and pointing its side chain towards the elbow helix (Fig. 2A). Indeed, E458R/A/D mutations completely abolished protein levels, ATPase activities, surface targeting, as well as mitoxantrone efflux (Fig. 2B–F). The results demonstrate that the glutamate is critical at position 458 for normal ABCG2 biogenesis. Further, D477, which precedes the putative kink in TMH3, is highly conserved among all mammalian ABCGs including yeast PDRs (Fig. S1B). D477A/E mutants exhibited normal protein levels, while D477R levels were strongly reduced. However, D477A/R lacked both mitoxantrone efflux and ATPase activity (Fig. 2B–D). Since D477E showed normal function, we believe that a negative charge at position 477 is essential for ABCG2 function (Fig. 2B–F).

### The elbow helix stabilizes the architecture of the transmission interface

**Figure 3:**
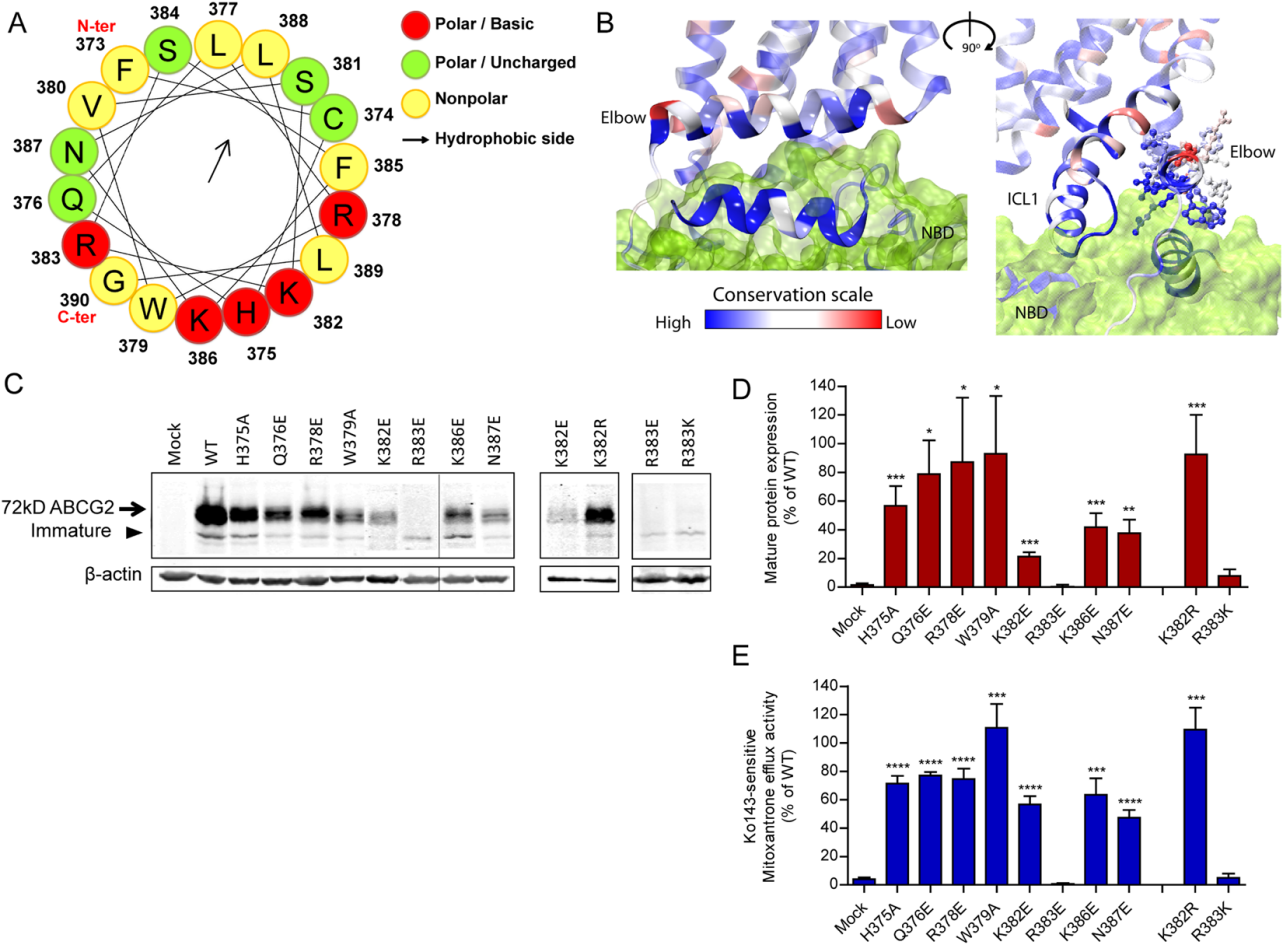
Functional analysis of the elbow helix. Residues in the elbow helix were subjected to mutational changes as indicated followed by their expression in HEK293 cells. (**A**) Elbow helix is depicted in a helical wheel projection using the HeliQuest analysis (http://heliquest.ipmc.cnrs.fr/) to reveal amphipathic properties. Residues are indicated in colored balls, charged/basic (red); polar (green); nonpolar (yellow); the arrow indicates orientation towards the hydrophobic membrane core (arrow). (**B**) Conserved motifs at the transmission interface of ABCG2 shown two views rotated by 90°. The degree of conservation among mammalian ABCGs is indicated as color-gradient; high conservation (blue) to low conservation (red). NBD surface (green). Side chains of elbow helix residues are represented as balls-and-sticks; residues not at atomic scale. (**C**) Immunodetection of ABCG2 variants after transfection into HEK293 cells. β-actin was used as an internal loading control. (**D**) Quantification of immunoblots from several independent experiments (n = 2–7), using the Odyssey system. ABCG2 signals are individually normalized to β-actin and shown as percentage of WT. The blots from different gels were divided by the white spaces. (**E**) Mitoxantrone efflux in HEK293 cells transfected with elbow helix mutants. Mitoxantrone accumulation was quantified in the presence and absence of Ko143 after incubation at 37 °C for 20 min. Ko143-sensitive mitoxantrone efflux is given as percentage activity relative to WT. Data are from several independent experiments (n = 2–7). All results are represented as means with SEM; ****P < 0.0001; ***P < 0.001; **P < 0.01; *P < 0.1 vs. empty plasmid-transfected HEK293 (mock).

The amphipathic elbow helix (F373 to G390) is likely to facilitate the anchoring of ABCG2 at the lipid/water interface (Figs 1 and 3A). Hence, the elbow helix may serve as a stable membrane-anchored hinge region between NBD and TMD, since it shares two interfaces with the upper part of NBD and ICL1 (Fig. 2A). Of note, the hydrophilic side facing the upper part of the NBD and ICL1 is highly conserved, while the membrane-exposed parts showed marginal conservation (Fig. 3B). Hence, we scrutinized residues including H375, Q376, R378, W379, K382, R383, K386 and N387 by mutational analysis. The single charge mutations demonstrated that R383E/K significantly impaired mitoxantrone efflux. This was perhaps due to reduced mature protein levels, although immature unglycosylated bands were still detectable. R383E/K also showed defective membrane sorting and accumulated intracellularly (Fig. S5). By comparison, H375A, Q376E, R378E, W379A, K382E, K386E and N387E retained the mature protein levels albeit at varying degrees, as well as mitoxantrone efflux Fig. 3C–E. Interestingly, K382E showing a severe expression defect while leaving function moderately unaltered. However, both expression and function were fully restored in the K382R mutant (Fig. 3C–E). All GFP-ABCG2 mutants (H375A, Q376E, R378E, W379A, K382E, K386E and N387E) showed normal surface localization. Taken together, our findings show and confirm a pivotal role of R383 in the elbow helix for normal ABCG2 folding, expression and membrane localization^46^.

### The transmission interface is stabilized by a salt bridge between elbow helix and ICL1

**Figure 4:**
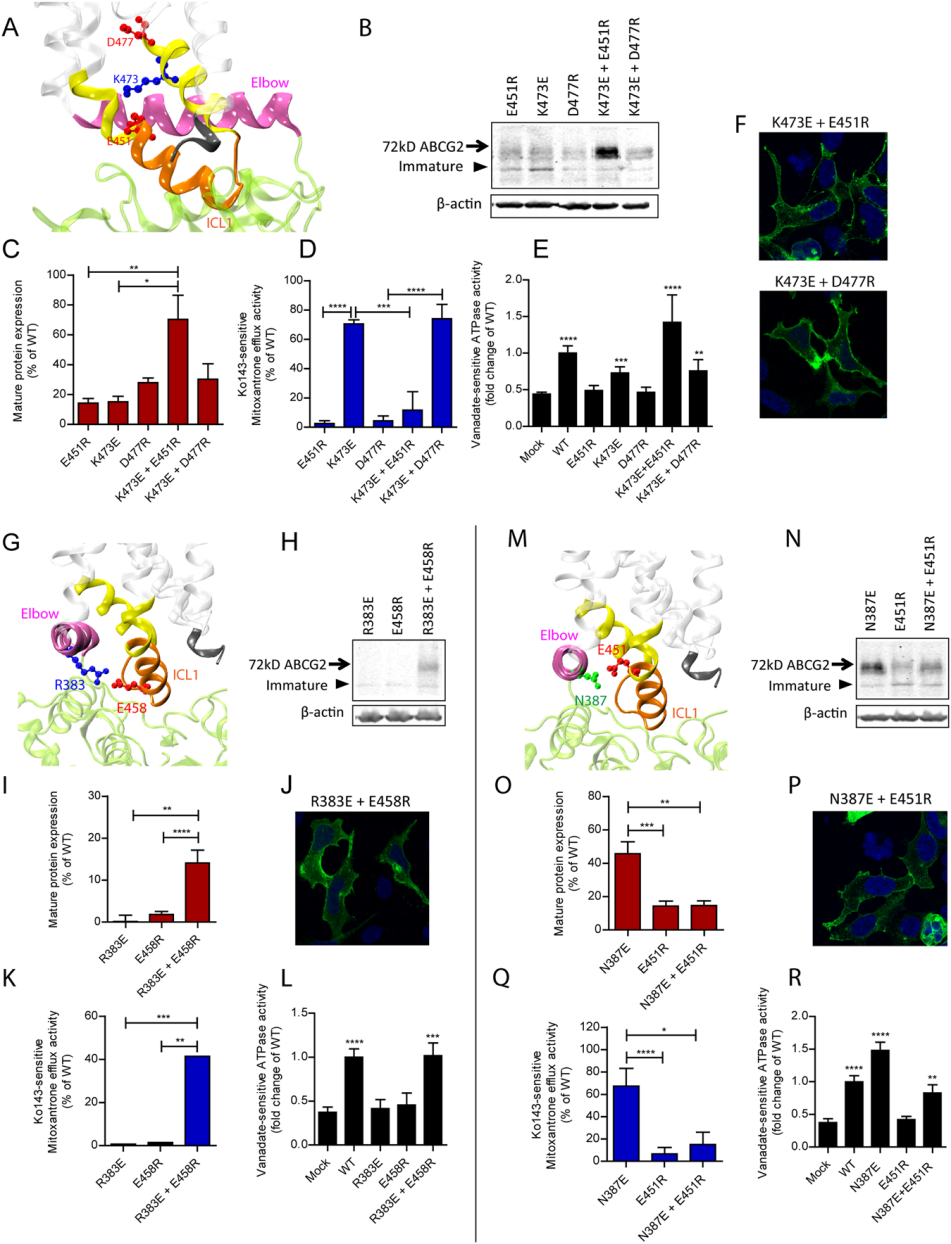
Interactions of charged residues at within transmission interface. Residues in the transmission interface were subjected to mutational changes as indicated followed by their expression in HEK293 cells. (**A**,**G** and **M**) Zoom-in views showing the side chains as balls-and-sticks with the positions of negative residues (red), positive residues (blue) and polar residues (green) in the transmission interface; ICL1 (orange); distal helical parts of TMH2 and TMH3 (yellow); TMHs (transparent white); elbow helix (pink); C-terminus (black); NBD ribbon (green). (**B**,**H** and **N**) Immunodetection of ABCG2 variants expressed in HEK293 using the anti-ABCG2 (BXP-21) antibody. β-actin was used as an internal loading control. (**C**,**I** and **O**) Quantification of normalized ABCG2 variants are from several independent experiments (n = 3). The blots from different gels were divided by the white spaces. (**D**,**K** and **Q**) Mitoxantrone efflux activity of ABCG2 variants are shown as percentage relative to WT from several independent experiments (n = 4–7). The bar graphs of expression and mitoxantrone efflux experiments were represented as means with SEM; ****P < 0.0001; ***P < 0.001; **P < 0.01; *P < 0.1. (**E**,**L** and **R**) Vanadate-sensitive ATPase activities of ABCG2 mutants expressed in HEK293 cells. Data are from several independent experiments (n = 7–8) using three batches of membrane preparations. Data are represented as fold change relative to WT. All means include SEM; ****P < 0.0001; ***P < 0.001; **P < 0.01 vs. empty plasmid transfected HEK293 (mock). (**F**,**J** and **P**) Membrane localization of GFP-tagged ABCG2 mutants was detected in the GFP channel (green). Nuclei were stained with DAPI (blue). Microscopy data are from representative duplicate experiments.

Several putative salt bridge interactions could stabilize the conformation the transmission interface. For example, K473 is close to E451 on the juxtaposed ICL1 and close to D477 in TMH3 (Fig. 4A). We hypothesized that E451 may form a salt bridge with K473. Indeed, single E451R and K473E mutations disrupted salt bridge formation. However, function was almost completely restored by combining both mutations E451R K473E in one transporter molecule, including normal surface localization of the GFP-ABCG2 K473E E451R variant (Fig. 4F). These data prove a functional salt bridge between E451 and K473 (Fig. 4B,C). Interestingly, the inverse double mutant E451R K473E was functionally impaired lacking mitoxantrone efflux (Fig. 4D). While surprising at first sight, these data reconfirm the essential role of E451 for ABCG2 function, since any mutation in this residue completely destroys substrate transport, yet maintains ATPase activity as seen for E451D (Fig. 4E). K473 and D477 reside on the same face of TMH3 and may therefore form a second salt bridge (Fig. 4A). While the single mutants D477R and K473E strongly decreased mature protein levels, the double mutation K473E D477R maintained low protein levels but restored both efflux and ATPase (Fig. 4B–E). D477R showed strongly impaired ATPase activity and mitoxantrone transport. Interestingly enough, adding K473E into D477R restored both ATPase (Fig. 4E) and mitoxantrone efflux (Fig. 4D). These data demonstrate that a negative charge next to K473 in TMH3 is essential for ATP hydrolysis and transport, though they are unlikely to form a functionally relevant salt bridge.

Furthermore, R383 in the elbow helix and E458 in ICL1 orient their side chains towards each other (Fig. 4G). Thus, a salt bridge may stabilize the interaction of the elbow helix with ICL1. The single mutations R383E or E458R completely destroyed expression, mitoxantrone efflux and ATPase activity (Fig. 4H–L). These results suggested that any mutation preventing salt bridge formation debilitates folding and consequently function. Interestingly, the double mutation R383E E458R significantly restored surface expression, including the recovery of mitoxantrone efflux and ATPase activity (Fig. 4H–L). These results demonstrate a vital salt bridge to exist between R383 in the elbow helix and E458 in ICL1.

The second putative interaction may connect N387 and E451 (Fig. 4M). Of note, both N387 and E451 are highly conserved (Fig. S1B). Both mutant variants N387E and E451R significantly reduced mature protein expression (Fig. 4N,O). Interestingly however, N387E showed strongly enhanced ATPase activity when compared to WT (Fig. 4R). The N387E E451R double mutant failed to rescue ABCG2 levels, although cell membrane targeting was detectable (Fig. 4N–P). However, N387E E451R almost fully regained ATPase activity (Fig. 4R), while mitoxantrone transport remained undetectable (Fig. 4Q). The results imply that N387 may interact with E451 though not via a typical salt bridge, although hydrogen bonds could still exist, whose disruption would impair ABCG2 function. Of note, when a negative charge replaces asparagine in N387E, the ATPase-dead phenotype of E451R was compensated in the E451R N387E double mutant, independently confirming a critical role for an interaction of N387 with E451.

### The interface between elbow helix and NBD dimer surface is crucial for protein folding

**Figure 5:**
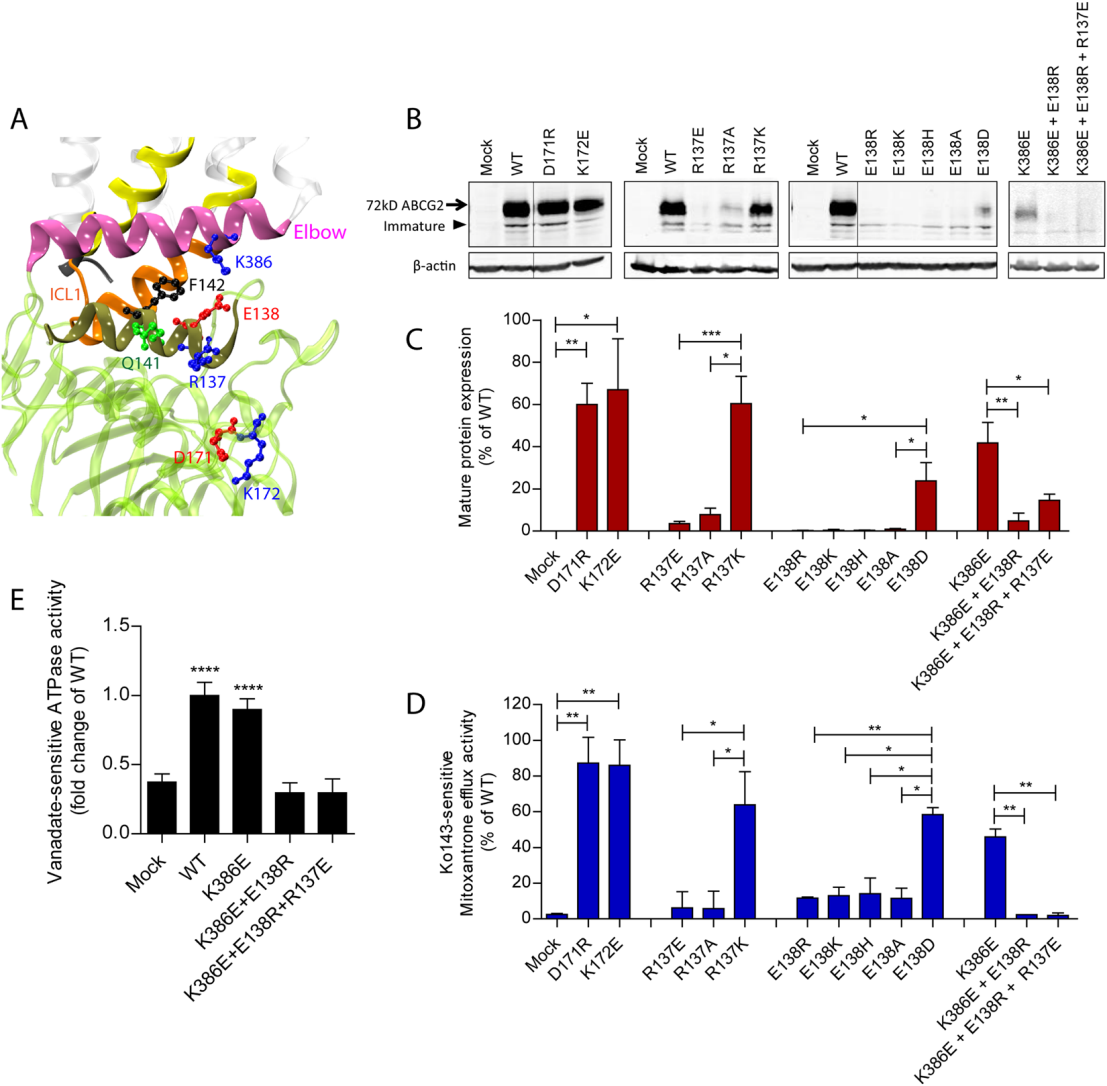
Charged residues at the NBD surface at the interface with the elbow helix are important for ABCG2 folding. (**A**) Zoom-in view of the interface between the elbow helix and the NBD (green). Elbow helix (pink); helical domain within NBD at the interface (dark green); ICL1 (orange); TMHs (transparent white); distal helical parts of TMH2 and TMH3 (yellow); C-terminus (black). The residue side chains are indicated as colored balls-and-sticks, positive charges (blue), negative charges (red), polar residues (green) and non-polar residues (black). (**B**) Immunodetection of ABCG2 mutants expressed in HEK293 cells, using β-actin as an internal control. The blots from different gels were divided by the white spaces. (**C**) Quantification of normalized ABCG2 levels was from several independent experiments (n = 2–6). Data are represented as a percentage relative to WT. (**D**) Mitoxantrone efflux activity of ABCG2 variants. Ko143-sensitive efflux activity are represented as a percentage activity of WT (n = 2–4). The bar graphs of ABCG2 level and mitoxantrone efflux are represented as means with SEM; ****P < 0.0001; ***P < 0.001; **P < 0.01; *P < 0.1. (**E**) Vanadate-sensitive ATPase activities of ABCG2 mutants. Data are from several independent experiments (n = 5–8) using three batched of membrane preparations. Data are presented as means with SEM; ****P < 0.0001 vs. mock.

The elbow helix orients in parallel to the inner leaflet of the membrane bilayer, facing residues R137 and E138 in the NBD. We refer to these charged residues as Region 1 (Fig. 5A). Subtending Region 1, residues D171 and K172 also reside on the NBD surface, to which we refer as Region 2. The highly conserved R137 and E138 putatively turn their side chains towards the elbow helix. Of note, they are located adjacent to Q141, the mutational hot spot in NBDs^47–51^. Conversely, D171 and K172 are not conserved. Indeed, D171R and K172E mutants maintained almost normal ABCG2 levels, including mitoxantrone efflux (Figs 5B–D and S5). These data suggest that Region 2 can in principle tolerate mutational changes.

By contrast, all of R137E/A and E138R/K/H/A mutations severely diminished mature ABCG2, as only immature bands were detectable (Fig. 5B,C). The surface delivery of the corresponding GFP-ABCG2 mutants was also undetectable and protein accumulated intracellularly (Fig. S5), explaining the apparent loss of mitoxantrone transport (Fig. 5D). However, mutants conserving the charge such as R137K and E138D maintained proper folding, localization and function (Fig. 5B–D). Therefore, these results unequivocally show that both R137 and E138 are essential for ABCG2 function, and, more importantly, a positive charge is required at position 137 and a negative charge must be present at position 138.

The side chain of K386 in the elbow helix is in close distance to Region 1 (Fig. 5A). We hypothesized about an interaction with Region 1 to stabilize protein folding. Thus, we generated double and triple mutants of K386E by adding the R137E and E138R mutations. Only the K386E single mutation partially retained ABCG2 transport function with full ATPase activity (Figs 5B-E and S5). However, combining Region 1 mutants with K386E to yield K386E E138R and K386E R137E E138R completely prevented mature ABCG2, membrane localization (Figs 5B–D and S5), ATPase activity as well as mitoxantrone efflux (Fig. 5D,E).

**Figure 6:**
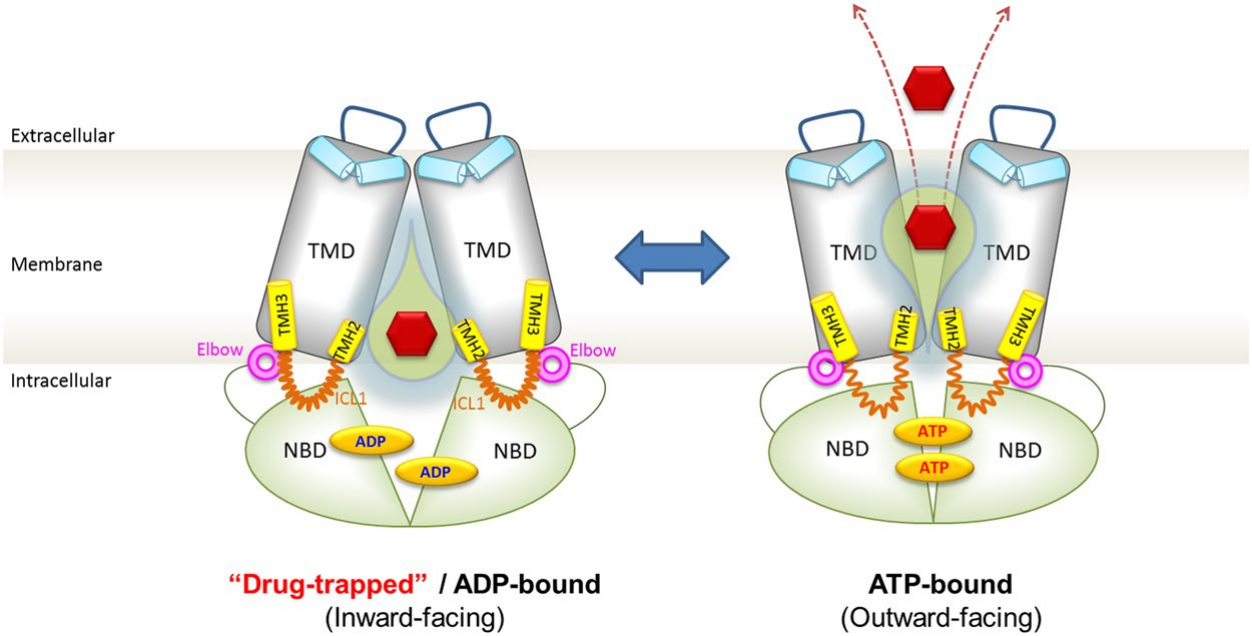
Hypothetical model depicting the ABCG2 transport cycle and the switch from the inward-closed to the outward-open conformation. The transmission interface, the NBD dimers (green), elbow helix (pink), distal parts of TMH2 and TMH3 (yellow) and ICL1 (orange), play a pivotal role in driving the conformational switch to the outward-open conformation for substrate (red hexagon) translocation. The re-entry helix (light blue), ECL3 (blue line), TMD (gray) and central cavity (yellow water drop). See main text for further details.

Taken together, the extensive mutational validation of the predicted atomic ABCG2 structure provided compelling evidence for a critical role of the transmission interface in the biogenesis, as well as the catalytic cycle and transport mechanism of ABCG2. Salt bridge interactions are essential to stabilize the architecture of the transmission interface. Moreover, the interactions of the elbow helix with ICL1 are crucial for ABCG2 function. Thus, the interfaces of ICL1-elbow helix-NBD are instrumental for the conformational switch to the outward-open drug-releasing conformation. Our data suggest that the transmission interface, and particularly ICL1, operates as a molecular spring by connecting ATP consumption at the NBD dimer with drug recognition in the central cavity. E451 plays an essential role in this process, as it serves as the central node of the transmission interface architecture (Figs 1 and 6). The switch to outward-open is guarded and stabilized by the interaction of ICL1 with the elbow helix, the latter providing a rotational axis for NBD and TMD movements. These interactions are essential for the catalytic cycle of ABCG2, the dynamic shape of the central drug cavity that accommodates numerous chemically distinct drug substrates. and explains the broad drug substrate specificity of ABCG2.

## Discussion

Here, we combine a molecular modeling approach with extensive molecular-genetic verification to validate a novel atomic structure of the human ABCG2 drug transporter. The mammalian ABCG family contains highly conserved structural domains, most of which are also present in yeast PDRs (Fig. S1). Hence, we reasoned that the recently reported crystal structure of ABCG5/G8^38^, the closest homologues of ABCG2, is the most suitable template to initiate a modeling approach for ABCG2. Moreover, this atomic model essentially corresponds to ABCG2 cryo-EM structure considering positions of both key domains and residues in the fold. This is not unexpected, since even the cryo-EM particles, owing to insufficient resolution in several regions^40^, were obtained by fitting cryo-EM densities on ABCG5, which is part of the ABCG5/G8 heterodimer. For example, residue R482^52,53^ as well as other key residues discovered in this report show identical positions in the atomic model and in the cryo-EM structure (Figs 1E and 5). While this is a striking independent conformation of the present model, it is important to note that neither the ABCG2 cryo-EM structure^40^ nor another homology model^41^ have been subjected to experimental verification to support structural predictions or mechanistic speculations. Moreover, older ABCG2 models used ill-posed templates, which, paired with the lack of proper validation, yielded incorrect structures or membrane topologies^54–56^.

The ABCG2 atomic model presented here shows a symmetric homodimer that adopts a compact fold with an apparent large central cavity. We speculate that the sites for substrate binding or trapping reside inside the central cavity, with the NBD dimer surface constituting the bottom lining of the central cavity. Moreover, the elbow helix, the re-entry helix^38^ and ICL1 are the most prominent and distinct structural elements (Fig. 1A). ABCG2 and ABCG5/G8 share the same fold, which is novel and unique among ABC exporters. Importantly, the distribution of polar residues across the TMDs in the ABCG2 transporter (Fig. 1), including the polar relay forming inside the TMD core^38^ is a novel functional element in the ABC transporter family. The polar relay may be less important for direct drug interaction, but very likely is critical for structural rigidity and stability (Fig. 1B,C).

We identify the transmission interface including one of the essential elements such as ICL1 as indispensable for both ATP consumption and drug efflux. In addition, we assign pivotal functions to individual charged ICL1 residues. In the ABCG family, ICL1 as part the transmission interface may be the equivalent of the “classical” coupling helix present in other ABC transporters. Remarkably, all mutations of E451 in ICL1 completely destroy ABCG2 function. However, mutational changes can lead to constitutively uncoupled ATPase activities, providing compelling evidence that drug binding/recognition and ATP hydrolysis may be independent and thus uncoupled events. Nonetheless, ICL1 is critical for the crosstalk of the catalytic ATP cycle with drug recognition, as well as for switching to the outward-open state. The contact of the kinked ICL1 with with the NBD surface (Fig. 5) strongly supports this notion.

The putative cytoplasmic loop after TMH2 and the C-terminus following TMH6 include eleven charged residues, several of which (K452, K453, R465) are dispensable for function. These data are consistent with earlier studies about the roles of K453, R465 and K652 mutants^44,57–60^. K473 may control dimer formation and maturation, since K473E impairs protein expression as reported earlier^44,47,57^. Interestingly, the structure places the three C-terminal lysines on the outer surface of the transporter, facing the lipid bilayer right at the border of the lipid-water interface (Fig. S7A,B). Interaction of these three lysines with phospholipid head groups may have a stabilizing effect on ABCG2, but does not have an critical role in transport. Indeed, mutational alterations of K647, K652 and K653 strongly reduce protein levels, but maintain mitoxantrone efflux (Fig. S7C–F). These data strongly suggest that the C-terminus may control the efficiency of folding, which is consistent with the fact that the core glycosylation patterns of immature precursors are distinct in all mutants (Fig. S7D).

Importantly, we here discover the critical importance of residue E446, which appears as the only charged residue facing the central cavity from TMH2. Most interestingly, E446 is not involved in ABCG2 folding, but strongly affects substrate recognition, consistent with previous reports^61^. We propose that E446 is part of the drug recognition zone in the central cavity, which is also critical to trigger substrate translocation through a drug channel as evident in the polarity distribution (Fig. 1). Of note, E451 locates just below E446, marking the beginning of the ICL1. E451 orients the side chain towards the elbow helix (Fig. 2A). Remarkably, any mutational change of E451 renders ABCG2 non-functional. Strikingly, and most unexpectedly, while E451D debilitates transport, basal ATPase activity is enhanced even in the absence of drug substrates (Fig. 2). We believe that the shorter aspartate side chain could interfere with the structural balance at the interface between ICL1, polar relay, elbow helix and NBD, possibly controling substrate recognition, but still allowing for enhanced ATP hydrolysis. Defects in these interactions may modulate the dynamics of the transmission interface such that ATP consumption is uncoupled from drug recognition, transport or even the conformational switch.

Interestingly enough, both E451 and E458 are extremely conserved among mammalian ABCGs though not in yeast PDRs (Fig. S1B). Mammalian ABCGs are all half transporters operating as homo- or heterodimers^62^, although evidence for ABCG2 tetramers exists^63–65^. By contrast, yeast PDRs operate as full transporters that do not require dimerization to form a functional efflux pump^26^. Indeed, both E451 and E458 are essential for ABCG2 function, since even conservative replacements such as in E458D debilitate protein folding, supporting a role of E458 in dimer formation (Fig. 2). Indeed, impaired homodimer formation of ABCG2 during or after translation exposes malfolded monomers to ERAD quality control^66^, resembling the scenario for yeast Pdr5, which is highly unstable when improperly folded in the ER membrane^67^.

Finally, D477 is also highly conserved in both mammalian ABCG and fungal PDR families. The structure places D477 into a helical stretch just before a kink in TMH3. D477 mutants are sorted to the surface, but removing the negative charge destroys function, suggesting a key role of D477 in the catalytic cycle. Of note, D477 is just one helical turn below of R482 in TMH3. R482 is a key residue controlling substrate specificity of ABCG2, since the R482G is a gain-of-function mutant^45,52,53,68,69^ that expands the drug spectrum of ABCG2, and perhaps, like D477, contributes to shaping the inner drug cavity. While the side chains of D477 and R482 may not be facing the central cavity, their positions within the polar relay indicates that mutations may affect structure, dynamics, and function of the substrate recognition.

We cannot entirely exclude that some mutations may alter the ABCG2 substrate or inhibitor specificity, especially when mutations change residues around the central cavity. For example, E446K/A/D and E451D show ordinary expression, membrane localization and retain ATPase activities but completely lack mitoxantrone efflux, as well as efflux of two additional ABCG2 substrates such as Rhodamine 123 and Hoechst 33342. Thus, mutations in E446 and E451 do not change substrate or inhibitor specificity.

The elbow helix constitutes a central element of the transmission interface. Remarkably, as in ABCG5/G8, it forms a horizontal amphipathic helix that may facilitate anchoring of the transporter to the inner leaflet (Fig. 3). The elbow helix may limit the conformational flexibility of ABCG2, and, more importantly, serve as a fixed hinge allowing for or supporting conformational changes and efficient energy transduction during the transport cycle. For example, our data show that the extremely conserved R383 is crucial for proper protein folding, which is in line with a previous report showing that R383 is vital for ABCG2 biogenesis^46^. Remarkably, K386 is juxtaposed to the upper part of the NBD in close distance to residues R137 and E138, which are immediate neighbors of Q141 and F142, both of which define a mutational hot spot in the NBD^47,70^. Q141 and F142 are important for NBD/TMD interaction and stabilize NBDs^47^. The gout-linked Q141K mutant impairs the stability of NBDs, whereas deletion of F142 affects dimerization^47^. The interface of the Region 1 (R137, E138, including Q141 and F142) with the elbow helix may be of vital importance to stabilize a symmetric conformation of ABCG2. Indeed, Region 1 is absolutely essential for protein folding and function, whereas Region 2 just beneath in the NBD is dispensable (Fig. 5).

The ABCG2 structure predicts that salt bridges within ICL1 could stabilize the conformation of the transmission interface or contribute to the architecture of the central cavity. This is reminiscent of vital salt-bridges verified in the corresponding ICL2 of human P-pg^71,72^. Indeed, the interaction of K473 and E451 are likely to stabilize ICL1, and perhaps ensure efficient coupling and energy transduction from NBDs via residue E451 (Fig. S3). Most surprisingly, the ATPase activity of the E451R K473E double mutant is even slightly increased, whereas mitoxantrone efflux is completely lost (Fig. 4), suggesting that the ATP cycle must not necessarily be coupled to drug transport in ABCG family members. Interestingly enough, yeast PDR efflux pumps act as constitutively “uncoupled” transporters, since they always hydrolyze ATP even in the absence of drug substrates^12,73^.

Furthermore, the model predicts two additional interactions connecting the elbow helix and the ICL1. Indeed, we unequivocally demonstrate a salt bridge between R383 and E458, since a double mutation R383E E458R recovers active transporter showing both ATPase activity and mitoxantrone efflux (Fig. 4H–L). Hence, the interaction between the elbow helix and the ICL1 are essential for ABCG2 biogenesis, while N387 may only interact with E451 via hydrogen bonds. Surprisingly, however, N387E shows enhanced ATPase activity, similar to increased ATP consumption displayed by in E451D in ICL1, the latter being close to the side chain of N387. Thus, at least one negative charge at the interface of E451 and N387 regulates ATPase activity and the dynamics of NBD interaction.

While the ABCG2 fold may be fully preserved in other ABCG and PDR family members, it will be worthwhile to test whether other members indeed share the same mechanism, since ABCG1, ABCG5/G8 and ABCG4 show a remarkably restricted sterol specificity. Noteworthy, the mechanism may be conserved in fungal PDRs, which act as both sterol transporters and classical polyspecific multidrug transporters^25^. Nonetheless, based on the atomic model of human ABCG2 and our comprehensive mutagenesis data, we wish to propose a mechanism for the catalytic cycle of ABCG2 (Fig. 6). We suggest that the ATP-free state adopts an inward-facing conformation, thereby enlarging the central cavity that may attract, recognize and trap drug substrates of remarkable structural and chemical diversity. Residue E451 is absolutely essential for transport, possibly by coupling ATP hydrolysis to substrate translocation or by regulating substrate binding. E446 in the middle of the TMD-TMD interface is the only charged residue facing into the central cavity. Mutational changes of E446 strongly support a role in substrate recognition. We speculate that the polar roof formed by extracellular re-entry helix including other extracellular loops (Figs 1 and 6) may acts as a barrier controlling substrate release channel or the switch to the outward-open state. The switch is executed by the transmission interface with a key role for ICL1 acting as a molecular spring. ATP-binding may drive NBD dimerization and trigger an upward movement of ICL1, which, in concert with the elbow helix triggers the conformational switch. This would also require the opening of the polar roof in the ECL to allow for substrate release, which might be tightly controlled by the re-entry helix. This notion is strongly supported by the “plug” proposed by the new ABCG2 cryo-EM structure to exist in this region^40^. Furthermore, the re-entry in ECL3 helix may play an pivotal role in substrate release, since it could act as a collar by interacting with ECL1 and ECL2 to form a plug or valve-like structure. Indeed, since several residues in ECL1-3 are indispensable for ABCG2 function (Khunweeraphong *et al*., in preparation).

Interestingly, ABCG2 may hydrolyze ATP even in the absence of drugs, which may or may not be accompanied by conformational switches. When drugs are trapped or recognized in the central cavity, for which E446 seems required, ICL1 couples the energy release at NBDs with TMDs, a process for which E451 is indispensable. In the inward-facing conformation, the ABCG2 maintains a central cavity, which is lined and stabilized by the polar relay in the membrane core (Figs 1 and 6). In the presence of drugs, ATP hydrolysis induces conformational changes in ABCG2 to the outward-facing conformation. In addition, ICL1 may also play a key role in regulating NBD dimer formation, supported by its interaction with the elbow helix. When drugs enter the central cavity from either the cytoplasm or laterally from the lipid bilayer, they are trapped requiring E446. The conformational switch is then enhanced by rigid movements of the transmission interface connecting the NBDs with TMDs. This requires a “clutch-like” function of ICL1 to achieve the coupling and the switch.

Further, we propose that attachment of the elbow helix to the inner leaflet of the membrane stabilizes the ABCG2 structure. Moreover, the elbow helix provides a hinge element, mechanically restraining conformational hyperflexibility of ABCG2. This scenario may restrict the conformational cross-talk between NBD and TMD to a simple rotational motion around a single axis. Owing to the particular configuration of the TMH2-ICL-TMH3 stretch, ICL1 could also serve as a molecular spring that controls the conformational equilibrium between the outward- and inward facing states depending on the presence of drugs and ATP. Based on our atomic model, it is tantalizing to speculate that the conformational switch creates a volume change in the substrate cavity and produces “pneumatic” effects during the switch, which concomitantly opens the polar roof or the plug for release. In other words, the cytosolic part narrows and thus becomes substrate-occluded, while the extracellular site opens allowing for the formation of the release channel (Fig. 6).

Posing a hen and egg problem, it is not clear at the moment whether ATP binding or hydrolysis drives NBD interaction or is resetting the cycle. The mutant data of the transmission interface clearly indicate that ATP binding and drug transport can be uncoupled and thus constitute in principle independent events. Hence, it remains open whether switching can occur in the absence of drugs when ATP is consumed. NBD dimerization may be constitutive demanding only the presence of ATP, which is plentiful under physiological conditions, as eukaryotic cells contain up to 5 mM concentrations of cytoplasmic ATP. Interestingly enough, none of the novel ABCG2 mutant variants discovered in this work have been found in clinical samples of cancer-related conditions or the genetics of gout.

In conclusion, this work details a feasible mechanism for the polyspecific human ABCG2 drug transporter. It discovers novel essential residues required for both biogenesis and ABCG2 function. The genetic data strongly support the structure and mechanism of catalytic cycle, which is distinct from any other eukaryotic ABC efflux transporter. Importantly, the proposed mechanism can answer key open questions about ABCG2 and thus may help to revamp efforts to therapeutically modulate ABCG2 in case of malignant or genetic diseases.

## Materials and Methods

Additional details on Materials and Methods references can be found in *SI Materials and Methods*. Supporting Information - SI Materials and Methods.

### Chemicals and Antibodies

Mitoxantrone, rhodamine-123, Ko143, ammonium molybdate, potassium antimony (III) tartrate hydrate, adenosine 5′-triphosphate disodium salt (ATP), ascorbic acid and ouabain octahydrate were purchased from Sigma-Aldrich (St. Louis, MO, USA). PEI (Poly-ethyleneimine) is obtained from Polysciences Europe (Eppelheim, Germany). G418 is from Santa Cruz Biotechnology (Dallax, TX, USA). Sodium orthovanadate is from New England Biolabs (Frankfurt, Germany). Oligonucleotides for site-directed mutagenesis were from Eurofins (Munich, Germany). Monoclonal mouse anti-ABCG2 (BXP-21) was purchased from Santa Cruz Biotechnology (CA, USA). Rabbit anti-β-actin (D6A8) was from Cell Signaling (Danvers, MA, USA). All other chemicals were of molecular biology grade from Sigma-Aldrich (St. Louis, MO, USA). Phusion DNA polymerase and DpnI are from NEB (New England Biolabs, MA, USA). Plasmids templates using in this study, pcDNA3.1(-)-hABCG2 (wild type) and pEGFPC1-hABCG2 (wild type) were kindly provided by Balázs Sarkadi (Institute of Enzymology, Research Centre for Natural Sciences, Budapest, Hungary).

### Plasmids construction and site-directed mutagenesis

All mutations were generated by using the pcDNA3.1(-)-hABCG2 or pEGFPC1-hABCG2 plasmids as templates. The site-directed mutagenesis was performed by using Phusion DNA polymerase, following the digestion of DNA templates by *DpnI* according to the manufacturer’s instructions, before transforming into *E*. *coli* (DH5α) for plasmid preparation and verification by DNA sequencing. The primers used in this study are indicated the Table S3 (see Supplemental Information).

### Cell culture and transfection

HEK293 cells were cultured in Dulbecco’s modified Eagle’s medium (DMEM) (Life Technologies, Rockville, MD, USA), supplemented with 10% FBS and maintained in the incubator at 37 °C, 5% CO_2_ with humidity. Transfection of HEK293 was performed using self-made poly-ethyleneimine (PEI) as previously described^74^. In brief, HEK293 cells were seeded onto a 6-well plate at a density of 5 × 10^5^ cells per well and cultured in DMEM (10% FBS) until cell reached 60–70% confluency. After 1-day of culturing, 2 µg of plasmid was mixed with 6 µl of 1 mg/ml of PEI (Poly-ethyleneimine) in 100 µl of Opti-MEM (Life Technologies, Rockville, MD, USA). The transfection reaction was incubated at room temperature for 15 min before dropping on to the cell culture. After 2 days post-transfection, cells were used for subsequent experiments. For stably expressing cell lines, HEK293 were generated as described^75^. After transfection for 48 h, cells were seeded at density range from 50–500 cells per plate into 10-cm dishes containing DMEM (10% FBS) supplemented 0.9 g/l of G418 as a dominant selectable marker. The culture medium was changed twice per week until the single colonies appeared. The single colony was isolated by trypsin digestion in cloning cylinder and transferred into 24-well plates. Cells maintained in G418 media for subsequent screening.

### Immunoblotting and protein methods

Cells were harvested by trypsin digestion, followed by single a washing step with ice-cold PBS. Cell pellets were lysed in protein lysis buffer containing 50 mM Tris (pH 8.0), 120 mM NaCl, 1 mM EDTA, 2% Triton X-100 and freshly added protease inhibitor cocktail (Halt Protease and Phosphatase inhibitor Cocktail, Thermo Scientific). Debris was removed by a brief centrifugation step at 1,200 × g for 2 min at 4 °C. The supernatants were mixed with the Laemmli sample buffer in the presence of freshly added 100 mM dithiothreitol (DTT) and subjected to SDS-PAGE. Western blot analysis was performed according to routine procedures. After electro-transfer of protein onto nitrocellulose blotting membranes of 0.45 μM pore size (GE Healthcare Life sciences, Freiburg, Germany), membranes were blocked for 1 h with 5% bovine serum albumin (BSA) in TBST buffer (TBS buffer containing 0.1% Tween-20), followed by incubation with the primary antibodies mouse anti-ABCG2 (BXP-21) or rabbit anti-β-actin (D6A8) at dilutions of 1:500 and 1:1000, respectively. After overnight incubation at 4 °C, membranes were washed three times with TBST buffer for 15 min each. Subsequently, the IRDye^®^ 800CW secondary antibodies (LI-COR Biosciences, Homburg, Germany) against mouse or rabbit were used for further incubation at room temperature for 1 h. Western blot signals were analyzed with the 800 channel of the Odyssey Imaging Systems and quantified by using Image Studio software ver2.1 (LI-COR^®^ Biosciences, Homburg, Germany). ABCG2 and β-actin were selected for quantification, and ABCG2 levels were normalized to β-actin before comparing with the expression level of wild type ABCG2 set as 100%. Data were analyzed from at least 3 independent experiments.

### Confocal microscopy

Cells were seeded onto cover glass in 24-well plates. After culturing for 2 days, cells were washed with ice-cold PBS and subsequently fixed with 4% formaldehyde in PBS at room temperature for 10 min. Slides were washed with PBS three times. Nuclei were stained by incubation with 5 µg/ml DAPI in PBS at room temperature for 5 min. After DAPI removal, cells were washed with cold PBS three times, and then incubated cells in 100 mM glycine for 15 min at room temperature. The cover slips were washed again in PBS before mounting on glass slides with mounting medium. Slides were dried in the dark and protected from light overnight before imaging cells in a Zeiss LSM700 confocal microscope using ZEN 2012 as analysis software.

### Protein stability analysis by cycloheximide chase assay

Stability of protein was performed by using cycloheximide to treat the cells at several time points to stop protein synthesis as described^76^. Briefly, stable cell lines expressing wild type and mutants-ABCG2 transporter were seeded in 24 well with 1 ml of DMEM (10% FBS, 1.5 mg/ml G418) and cultured for overnight. After remove medium, cells were treated with cycloheximide (Sigma-Aldrich, St. Louis, MO, USA) at final concentration 100 µg/ml (containing 0.1% DMSO) for 0, 1, 3, 6, 12 and 24 h. by using 0.1% DMSO treatment as a control. All samples were collected at the same time and harvested by centrifugation at 15,000 g for 1 min at 4 °C. Cell pellet was lysed in protein lysis buffer containing freshly added protease inhibitor cocktail before perform western blot analysis with mouse anti-ABCG2 (BXP-21) or rabbit anti-β-actin (D6A8) as procedure described above. Quantification used the Image Studio software ver 2.1 (LI-COR^®^ Biosciences, Homburg, Germany). Regions of measurements for both ABCG2 and β-actin were selected.

### Membrane protein preparation

Membranes were prepared as previously described^45^. In brief, HEK293 cells were seeded and transfected with ABCG2 constructs for 3 days. Cells were washed with ice-cold PBS twice before harvesting. Cell pellets were resuspended in ice-cold TMEP buffer (50 mM Tris pH7, 50 mM Mannitol, 2 mM EGTA and protease inhibitor cocktail), and lysed by passing the suspension for 20times through a 27-gauge needle using a syringe. Debris were removed by centrifugation at 500 × g for 10 min; mitochondria proteins was sedimented by centrifugation at 1,200 × g for 5 min. Supernatants were subjected to untracentrifugation at 100,000 × g for 60 min. Membrane pellets were resuspended in TMEP buffer containing protease inhibitor and adjusted to a protein concentration of 2 mg/ml. All procedures were always performed at 4 °C. The membrane proteins were aliquoted and stored at −80 °C. The protein concentration was measured by Bradford assay.

### ATPase assay

The vanadate-sensitive ATPase activity of ABCG2 in transfected HEK293 membrane protein was quantified by established procedures^77,78^ with minor modifications. Briefly, 5 µg of total membranes were preincubated in ATPase assay buffer (50 mM MOPS, 50 mM KCl, 0.5 mM EGTA, 5 mM NaN_3_, 2.5 mM DTT, 1 mM Ouabain, pH 7) in the presence or absence of 100 µM sodium orthovanadate at 37 °C for 10 min. The reaction was started by addition of 4 mM of ATP/Mg^2+^ in a total volume of 50 µl. After incubation at 37 °C for 30 min, 40 µl of 5%SDS was added to stop the reactions. Subsequently, 100 µl of color reagent containing 3.33% (v/v) H_2_SO_4_, 0.48% (w/v), ammonium molybdate, 0.006% (w/v), antimony potassium tartrate, 5.7% (v/v) acetic acid and 0.24% (w/v) ascorbic acid (freshly prepared) was added and incubated at room temperature for 30 min. The released inorganic phosphate was examined by colorimetric assay at 800 nm in a microplate reader (Victor, Perkin Elmer, Turku, Finland). The SDS-treated sample was prepared in parallel as a background control. A phosphate standard curve was used for calculation. The vanadate-sensitive ATPase activity was calculated by subtracting from vanadate-treated sample.

### Flow cytometry

Flow cytometry was used to examine the transport activity by quantifying the intracellular steady-state accumulation of ABCG2 substrates such as mitoxantrone or rhodamine-123 in HEK293 cells in the presence or absence of the specific ABCG2 inhibitor Ko143, as described before^56,79–82^. In brief, cells were trypsinized and harvested in DMEM (10% FBS) before washing in ice-cold PBS. The Cell pellets containing 10^5^ cells per data point were resuspended in 25 µl of HPMI buffer pH7.4 (10 mM Hepes, 120 mM NaCl, 5 mM KCl, 400 µM MgCl_2_, 40 µM CaCl_2_, 10 mM NaHCO_3_, 10 mM glucose, 5 mM Na_2_HPO_4_). The cells were pre-incubated with 25 µl of 2% DMSO (as DMSO control) or 25 µl of 4 µM of Ko143 (containing 2% DMSO) for 5 min at 37 °C, before addition of 50 µl of 14 µM of mitoxantrone to a final concentration of 7 µM in 100 µl of reaction volume. After incubating for further 20 min at 37 °C, transport was stopped by putting the tube into ice-cold water for 5 min, followed by washing cells with 500 µl of ice-cold PBS and centrifuged at 600 × g for 2 min at 4 °C. After removing supernatants, cell pellets were resuspended in 150 µl of ice-cold PBS before subjecting to analysis using the BD FACSCalibur (Becton Dickinson, San Jose, CA, USA), with FL3 at the excitation and emission at 488 and 670 nm, respectively. For rhodamine-123 efflux, 50 µl of 1 µM of rhodamine-123 was added to yield a final concentration at 0.5 µM and assayed as described for mitoxantrone but determined with FL1 at the excitation and emission wavelengths at 488 and 534 nm, respectively. Viable cells were gated for populations from forward and side scatter. Ten thousand of cells were subjected for analysis of each data point by using FlowJo Software Inc. (Stanford University). The intensity of fluorescence in each sample was corrected from unstained cells as a background. Data were normalized to the activity in the presence of the Ko143 inhibition, which was set as 100% inhibition. The results are represented as percentage of activity of wild type ABCG2. When 5 µg/ml of Hoechst 33342 dye was used as a substrate, we used the same conditions as for mitoxantrone or rhodamine-123. Measurements were carried out using the FACS Aria (Becton Dickinson, San Jose, CA, USA) with the excitation and emission at 355 and 424 nm, respectively.

### ABG2 homology modeling and electrostatic potential calculations

We created 50 homology models of the human ABCG2 transporter in the ATP-free state using Modeller, version 9.16^83,84^ using the crystal structure coordinates of the heterodimeric ABCG5/G8 (PDB ID: 5DO7)^38^ as a template. Structure-based sequence alignments of ABCG2, ABCG5 and ABCG8 were used. All models were sorted by their discrete optimized protein energy (DOPE) score. The best model resembling the structure of ABCG5/G8 was selected for analysis.

### Data and statistical analysis

All values in this study are represented as mean + SEM. unless stated otherwise. All *in vitro* experiments were performed using at least 3 independent biological replicates. In case of published ABCG2 mutant variants, quantification of efflux activities and expression levels were repeated only twice to recapitulate and confirm published phenotypes. Statistical analyses were performed by using an unpaired t-test using the GraphPad Prism Software (San Diego, CA, USA) version 5.00.

### Data availability

The authors confirm that all data underlying the findings are fully available without restrictions. All relevant data are within the paper and the Supplementary information.

## Electronic supplementary material

Supplementary Information
