# Supplementary material for: The structure of the human ABC transporter ABCG2 reveals a novel mechanism for drug extrusion
Source: Sci Rep. 2017 Oct 23;7:13767. doi: 10.1038/s41598-017-11794-w (PMC5653816; doi:10.1038/s41598-017-11794-w)
Supplement: Supplementary file 1 — Supplementary Information [file 41598_2017_11794_MOESM1_ESM.doc]

**Supplemental Information – Figures & Legends**

**The structure of the human ABC transporter ABCG2 reveals**

**a novel mechanism for drug extrusion**

Narakorn Khunweeraphong^1^, Thomas Stockner^2^ & Karl Kuchler^1,a^

^a^ **To whom corresponding may be addressed:**

Karl Kuchler, Medical University of Vienna, Center for Medical Biochemistry

Max F. Perutz Laboratories, Campus Vienna Biocenter, 1030 Vienna, Austria

e-mail: [karl.kuchler@meduniwien.ac.at](javascript:linkTo_UnCryptMailto('kygjrm8iypj,isafjcpYkcbslgugcl,ya,yr');)

Phone: +43-1-4277-61807; FAX: +43-1-4277-9618

**
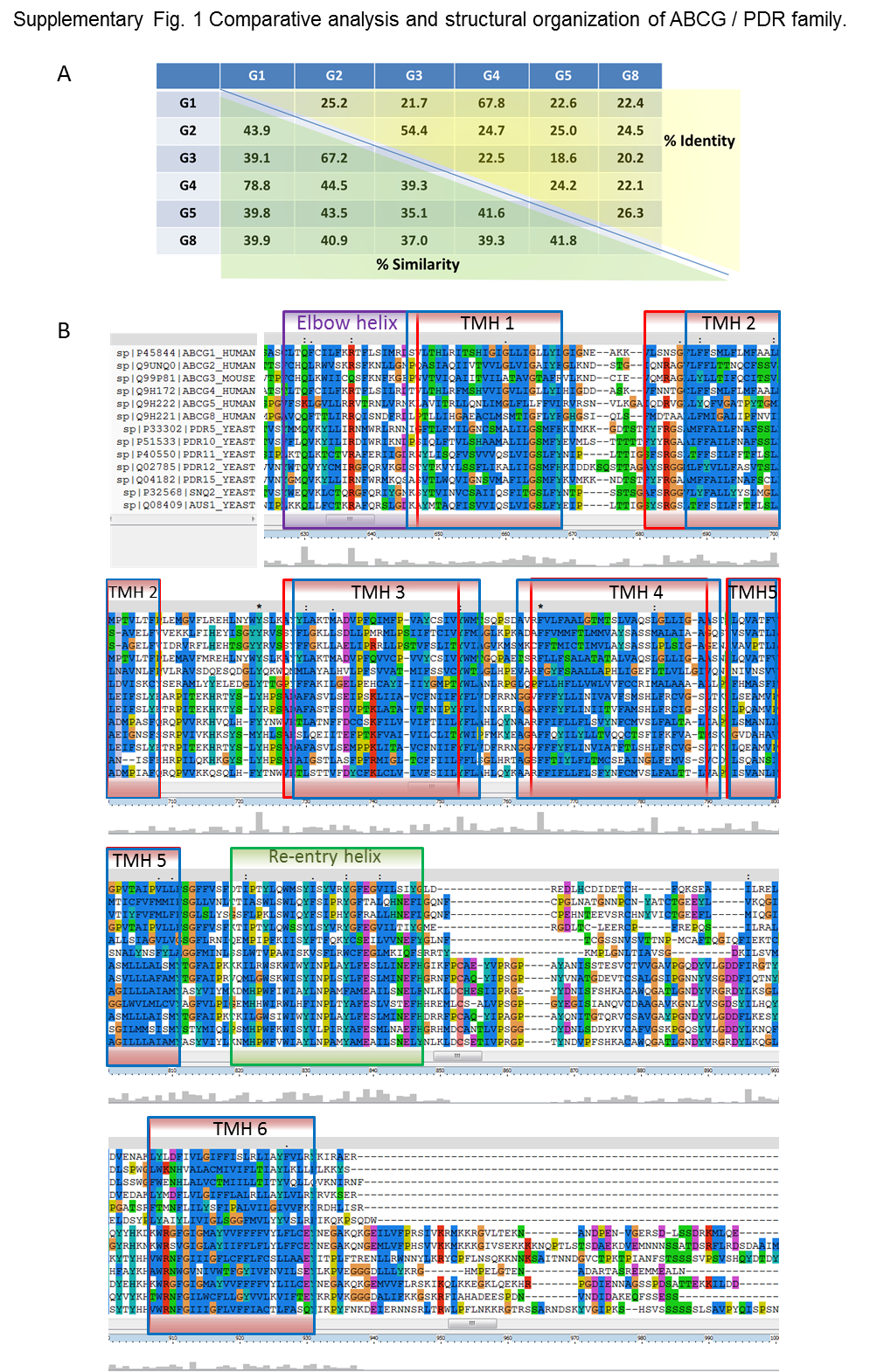
**

**Supplementary Figure 1**

**Comparative analysis and structural organization of ABCG / PDR family.**

(**A**) Pairwise alignment analysis of the mammalian ABCG family. The pairwise alignment of the primary sequences of human ABCGs using Vector NTI indicates the percentage of identity (yellow-shaded) versus similarity (green-shaded) for each pair of human ABCGs transporter. (**B**) Primary sequence alignments of mammalian ABCGs and the first half of yeast PDRs. Sequence alignments from TMD regions of mammalian ABCGs and the first half of yeast PDRs were conducted using ClustalX2. The conserved residues are highlighted with the conservative scale in each position (height of grey bars at the bottom). The elbow helix and re-entry helix are marked in violet and green boxes, respectively. The putative membrane-spanning helices TMH1-6 based on the ABCG5/ABCG8 structure are indicated by boxes, ABCG5 (red line), ABCG8 (blue line).


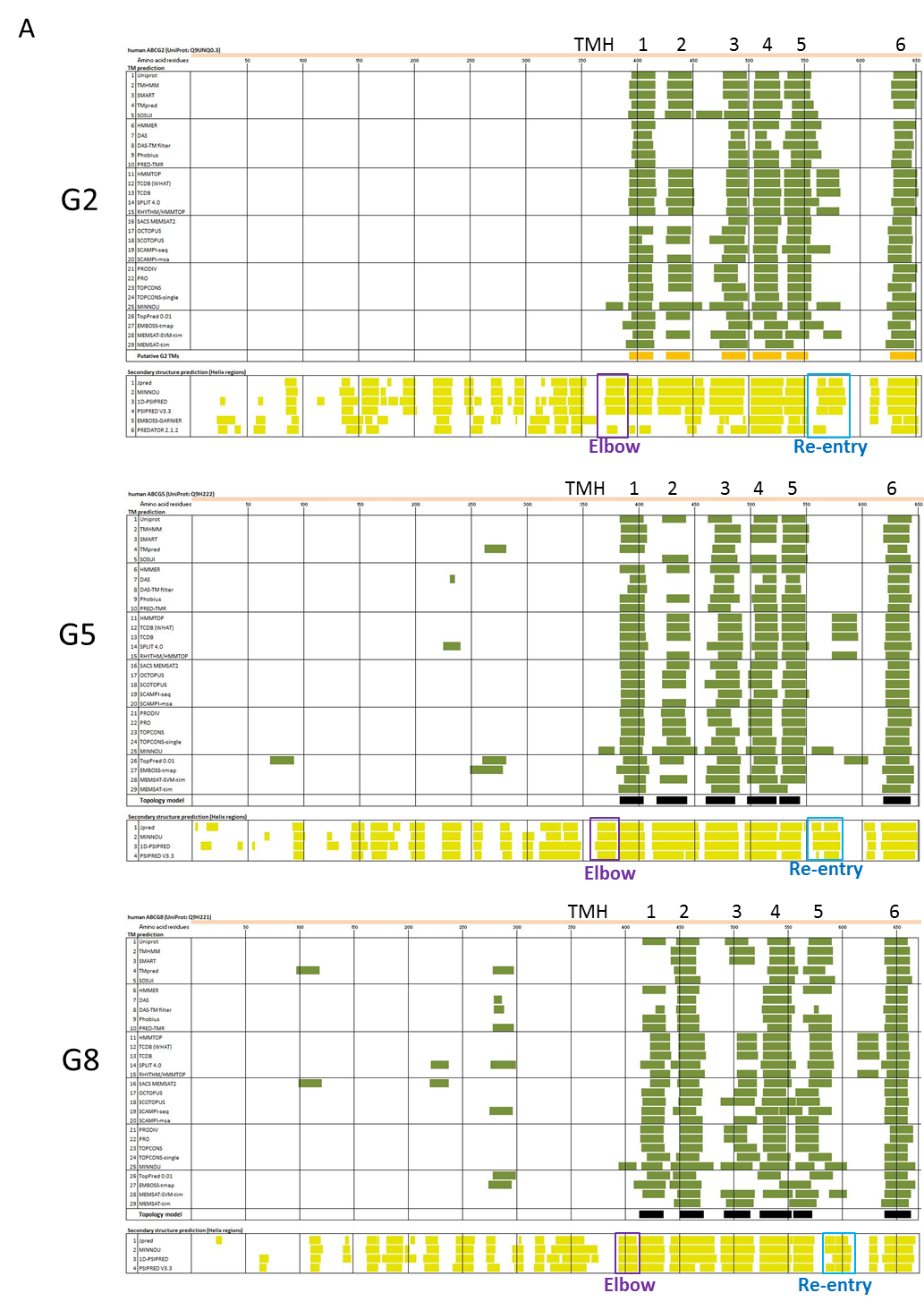


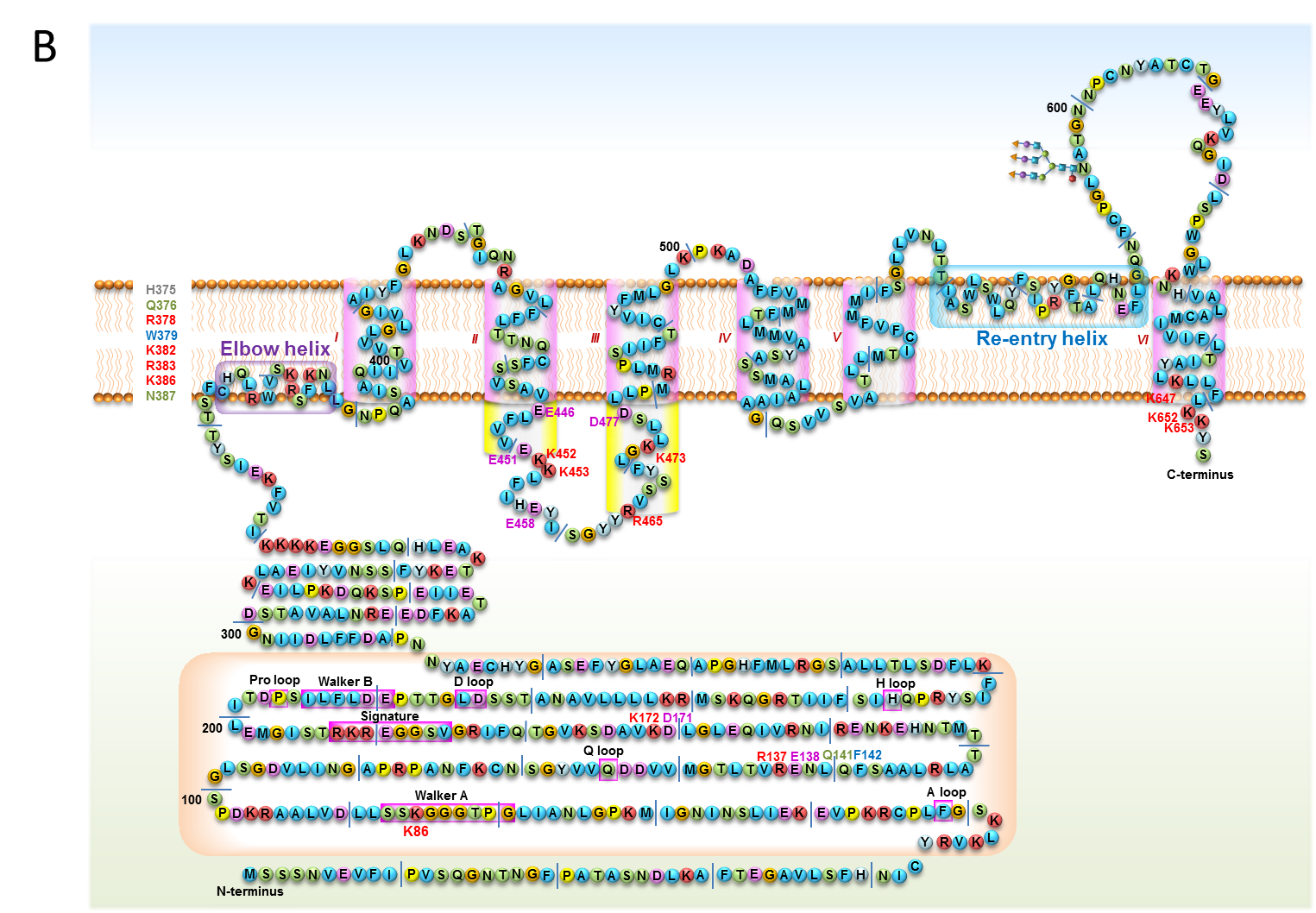


**Supplementary Figure 2**

**Transmembrane-spanning helices (TMH) and secondary structure prediction of ABCG2, ABCG5 & ABCG8.**

(**A**) Transmembrane-spanning helices (TMH) and secondary structure prediction of ABCG2, ABCG5 & ABCG8. The output by prediction tools for full-length human ABCG2 (Q9UNQ0, 655 amino acids), ABCG5 (Q9H222, 651 amino acids) and ABCG8 (Q9H221, 673 amino acids) are aligned. Putative TMH regions are marked in green bars. Helical regions are represented in yellow bars. Putative TMH regions of ABCG2 are depicted as orange bars. The TMH regions based on the topology of human ABCG5/G8 are shaded in black. The violet box indicates elbow helix, and the light blue box the re-entry helix. (**B**) Predicted membrane topology configuration of the human ABCG2 half transporter. The predicted membrane topology of ABCG2 was generated based on TMH prediction and secondary structure analysis. The NBD is highlighted in the orange box with highly conserved regions in pink boxes. A-loop, Walker A, Q-loop, signature motif, Pro loop, Walker B, D-loop and H-loop, respectively. The elbow helix precedes the TMH1 at the cytoplasmic lipid/water border (violet box). The external re-entry helix (light blue box) after TMH5 in ECL3 is in the outer leaflet of the lipid bilayer. Properties of amino acid residues are represented as color-coded balls; hydrophobic (blue), small hydrophobic (orange), proline (yellow), amphipathic (white), polar (green), positively charged (red) and negatively charged (pink). The consensus glycosylation site is indicated at N596. The charged resides subjected to mutagenesis her are indicated; positive residues (red); negative residues (pink). The yellow cylinders indicate the TMH regions derived from the homology model of ABCG2 based on ABCG5/G8 crystal structure.


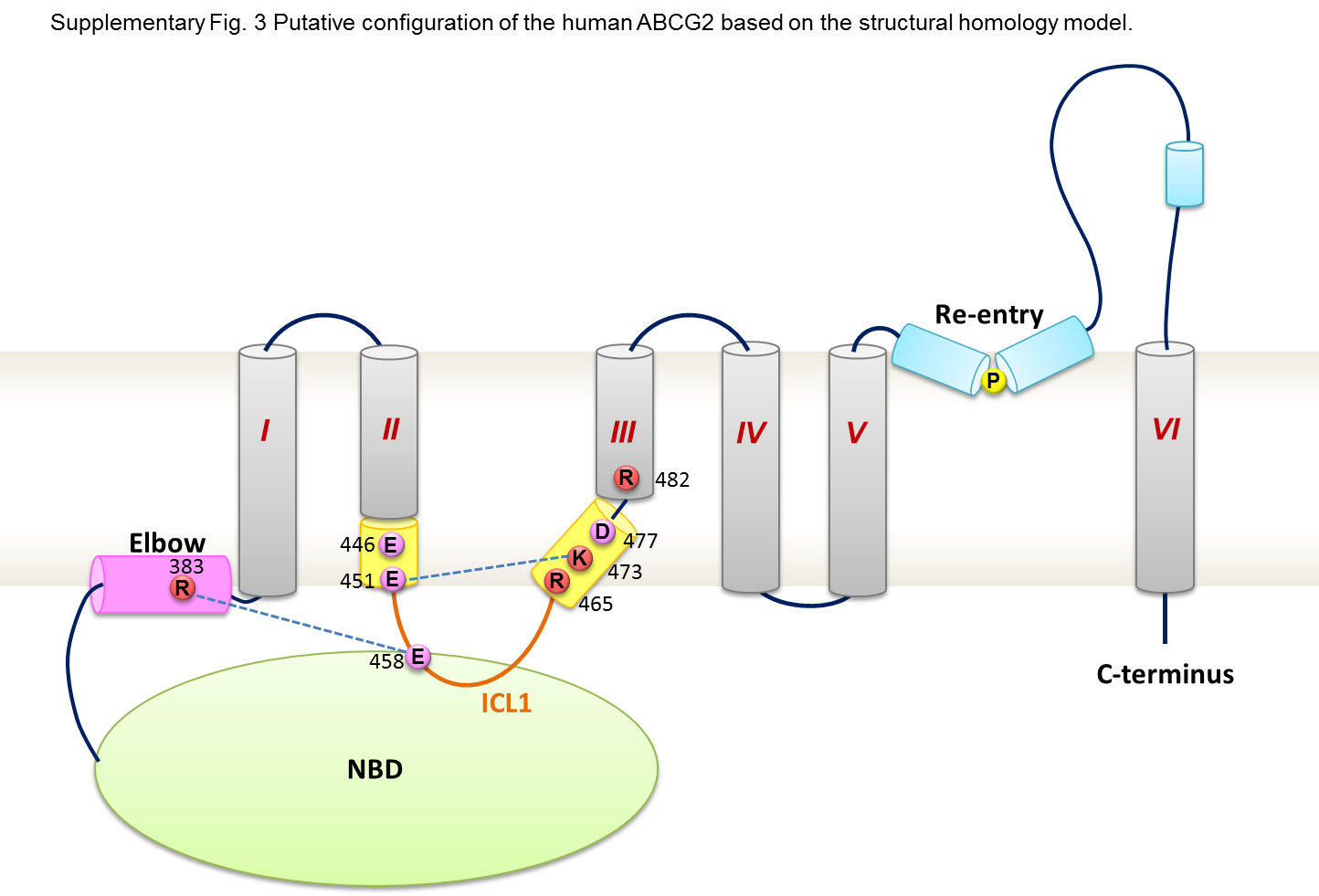


**Supplementary Figure 3**

**Putative configuration of the human ABCG2 based on the structural homology model.**

The configuration of ABCG2 indicates the N-terminus NBD (green). Helical features are represented as colored cylinder; elbow helix (pink), 6 TMH1-6 (grey), ICL1 (orange), re-entry helix (light blue). Amino acid residues are shown as colored balls, negative (pink) and positive (red). Proline is in yellow. The salt bridge interaction between E451 and K473 of the ICL1 and the interaction of R383 from elbow helix with E458 in ICL1 are indicated by dotted lines. ICL1 begins at residue E451 and ends at R465 to connect TMH2 and TMH3 in the cytoplasm.


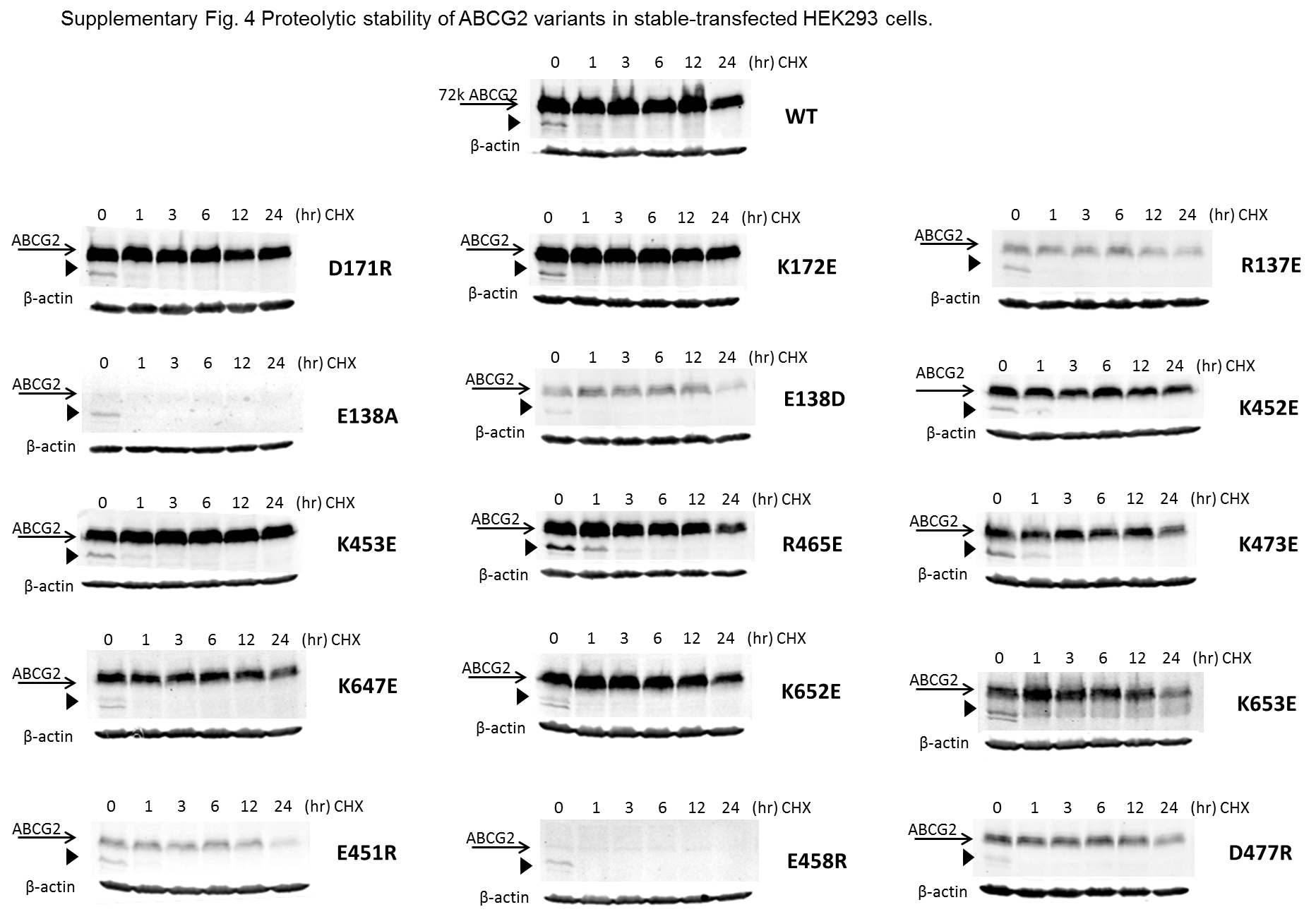


**Supplementary Figure 4**

**Proteolytic stability of ABCG2 variants in stable-transfected HEK293 cells.**

Cells were cultured in 24-well plates overnight; cells were treated with cycloheximide to stop protein translation for the indicated time points. Total cell-free lysates were prepared and subjected to immunoblotting using the monoclonal anti-ABCG2 (BXP-21) antibody. Arrow, mature glycosylate protein at approximately 72 kDs whereas the arrow head shows the immature bands. β-actin was used as an internal loading control.


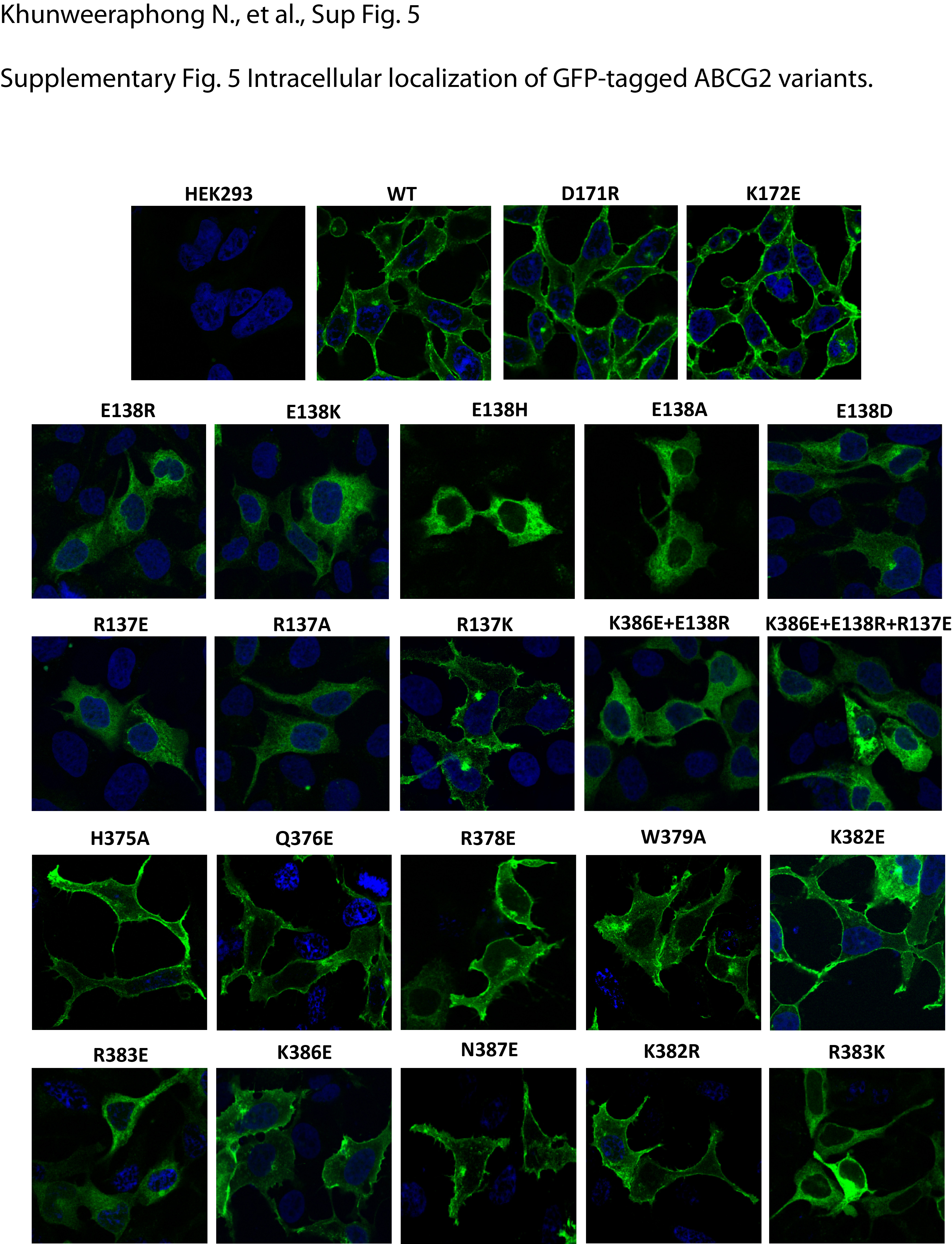


**Supplementary Figure 5**

**Intracellular localization of GFP-tagged ABCG2 variants.**

GFP signals of GFP-tagged ABCG2 were recorded in a confocal microscope. The signal observed on the plasma membrane in the presence of mature protein whereas the signal from immature proteins are located in the intracellular parts. DAPI was used as nuclei staining and show in blue signal. The results were conducted in duplicate and represented the same patterns.

**
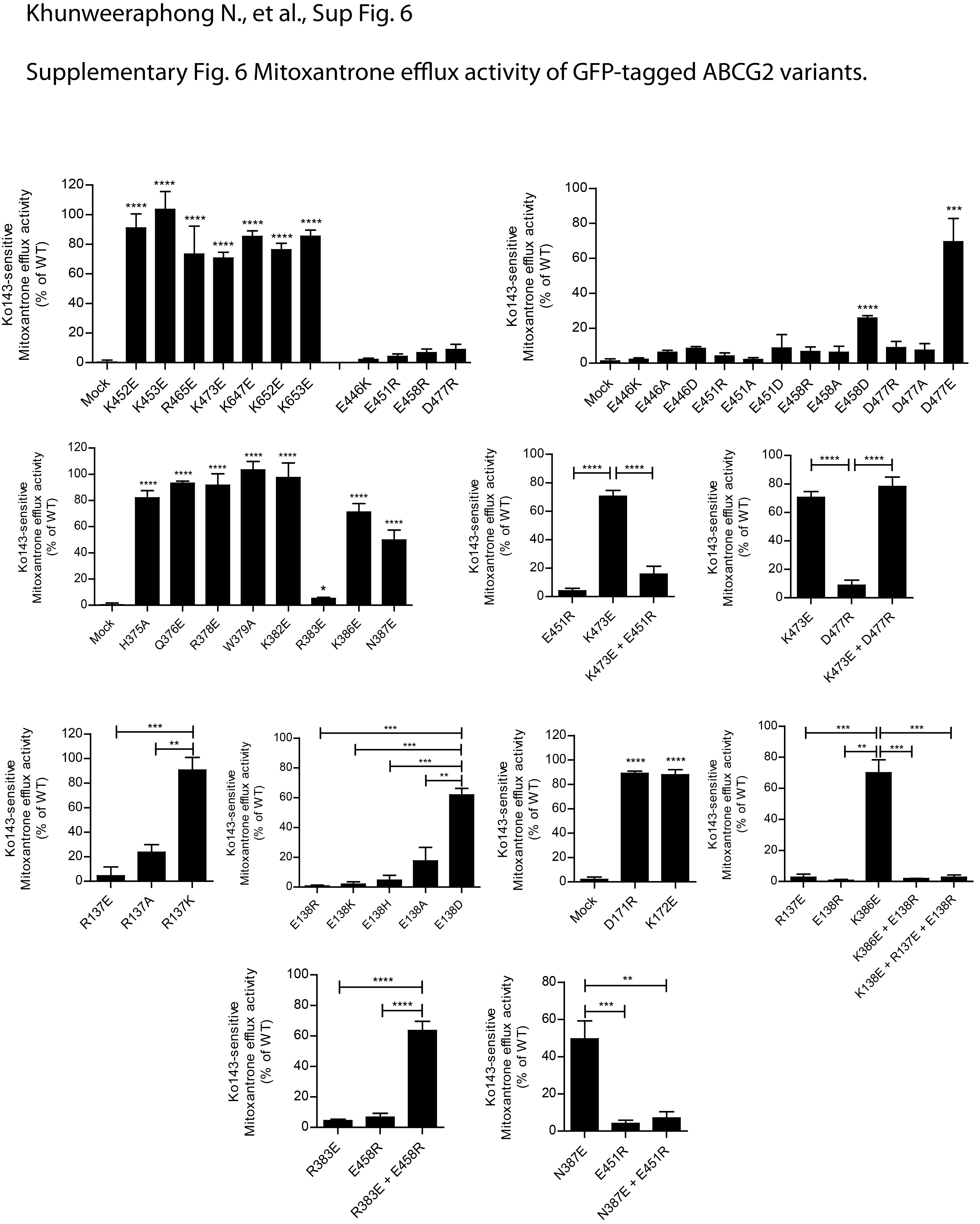
**

**Supplementary Figure 6**

**Mitoxantrone efflux activity of GFP-tagged ABCG2 variants.**

Mitoxantrone efflux of GFP-tagged ABCG2 mutants was used to verify function of ABCG2-GFP variants transfected into HEK293 cells. Intracellular mitoxantrone accumulation was determined in the presence and absence of the ABCG2 inhibitor, Ko143. GFP-expressing cells were gated for fluorescence intensity calculation. The experiments were performed at least twice. Data are presented as means with SEM, (n = 2-6); *****P* < 0.0001, ****P* < 0.001, ***P* < 0.01 and **P* < 0.1 vs. mock control, otherwise indicated as bar lines.


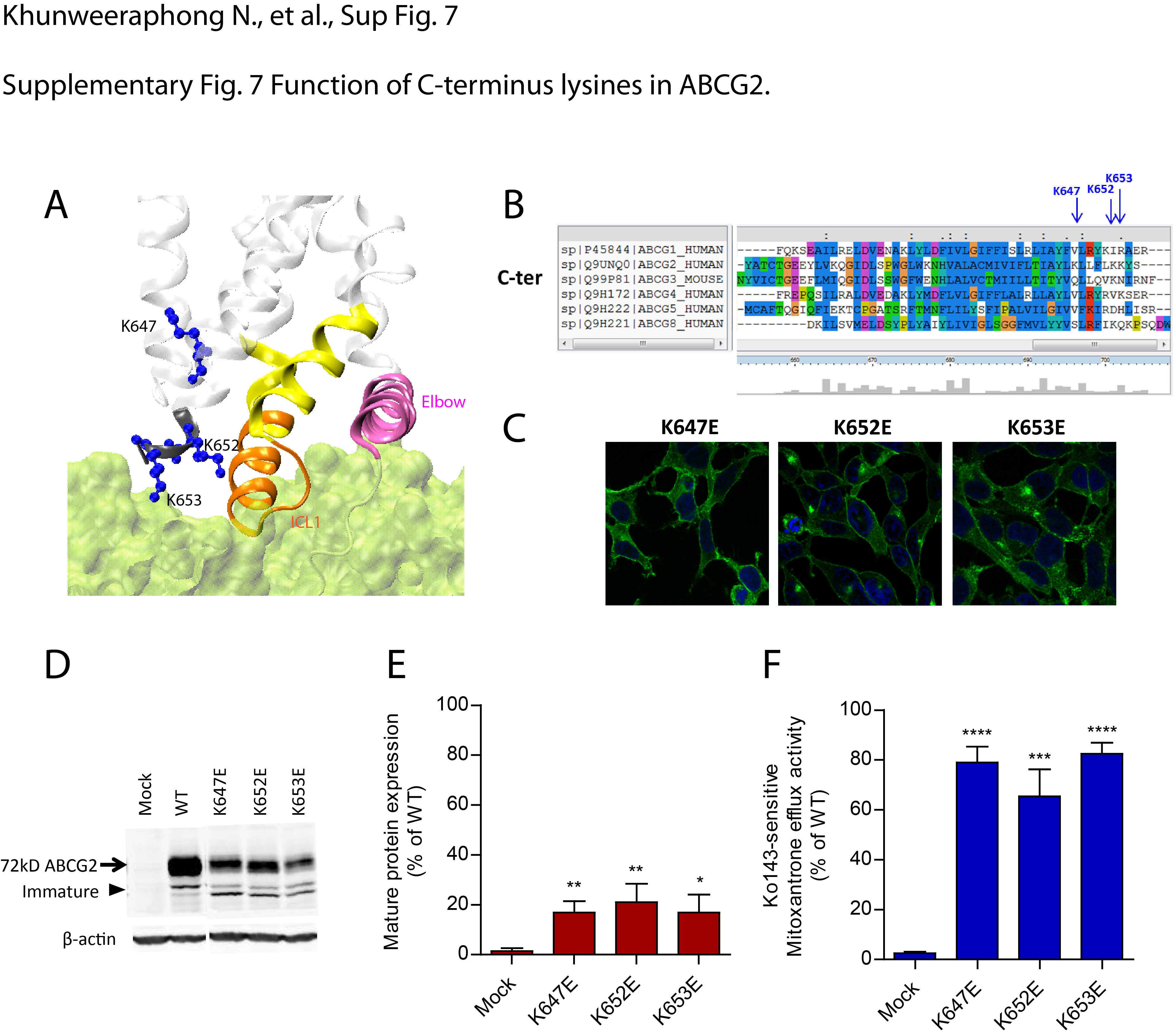


**Supplementary Figure 7**

**Function of C-terminal lysines in ABCG2.**

(**A**) Zoom-in view showing positions of residues K647, K652 and K653 at the C-terminus of ABCG2 juxtaposed to ICL1 (orange); distal helical parts of TMH3 and TMH2 (yellow); elbow helix (pink). Side chains of lysine residues in balls-and-sticks (blue), NBD surface (green), TMHs (transparent white), C-terminus (black). (**B**) Amino acid alignments of mammalian ABCGs. Sequences alignment from the C-terminus of mammalian ABCGs was performed by ClustalX2. The conserved residues are highlighted with the conservative scale bar (grey) of each position at the bottom. The positive charged residues K647, K652 and K653 of ABCG2 are indicated with the blue arrows. (**C**) Localization of GFP-tagged ABCG2 variants with mutations in the C-terminus. Signals of GFP-tagged ABCG2 were detected in the GFP channel (green). Nuclei were stained with DAPI (blue). Data are derived from representative duplicate experiments. (**D**) Immunodetection of ABCG2 variants in cell-free lysates from HEK293 transiently transfected with mock, wild type control (WT) and ABCG2 variants using the monoclonal mouse anti-ABCG2 (BXP-21) antibody. β-actin expression was used as a loading control. (**E**) Quantification of normalized ABCG2 levels from several independent experiments (n=3-5). Data are presented as means with SEM; ***P* < 0.01; **P* < 0.1 vs. mock. (**F**) Ko143-sensitive mitoxantrone efflux activity of C-terminal ABCG2 mutants represented as means with SEM. Data are means from several independent experiments (n=3-5). *****P* < 0.0001; ****P* < 0.001 vs. empty vector control (mock).

**Supplemental Information – Tables**

**Supplementary Table 1 Predicted transmembrane helices (TMH) in human ABCG2.**

The weblinks of online sources for tools are listed. The amino acid residue position corresponding to TMHs are given relative to start methionine.

**Programs Website Links TMH1 TMH2 TMH3 TMH4 TMH5 TMH6 TMH7**

1 Uniprot [www.uniprot.org/uniprot/Q9UNQ0,3](http://www.uniprot.org/uniprot/Q9UNQ0,3) 396-416 429-449 478-498 507-527 536-556 631-651

2 TMHMM predict www.cbs.dtu.dk/services/TMHMM 394-416 428-450 478-499 506-528 533-555 629-651

3 SMART smart.embl-heidelberg.de/ 394-416 428-450 478-499 506-528 533-555 629-651

4 TMpred www.ch.embnet.org/software/TMPRED 396-416 429-450 483-499 505-530 540-558 631-649

5 SOSUI bp.nuap.nagoya-u.ac.jp/sosui/ 393-415 426-448 454-476 479-500 506-528 540-562

6 HMMER hmmer.janelia.org/ 396-416 483-499 505-527 539-565 631-650

7 DAS www.sbc.su.se/~miklos/DAS/ 398-413 485-496 507-516 534-560 632-647

8 DAS-TMfilter mendel.imp.ac.at/sat/DAS/DAS 397-414 483-497 507-520 532-562 632-648

9 Phobius phobius.sbc.su.se/ 396-416 483-499 505-527 539-565 631-650

10 PRED-TMR athina.biol.uoa.gr/PRED-TMR/ 399-416 483-499 506-527 539-556 629-646

11 HMMTOP www.enzim.hu/hmmtop/ 394-416 429-450 481-499 506-528 533-555 562-581 631-651

12 TCDB (WHAT) www.tcdb.org 394-416 429-450 481-499 506-528 533-555 562-581 631-651

13 TCDB www.tcdb.org/progs/TMS.php 394-417 429-451 481-500 506-529 533-556 562-582 631-652

14 SPLIT4.0 split.pmfst.hr/split/4/ 393-415 427-451 482-498 505-528 533-563 626-649

15 RHYTHM/HMMTOP proteinformatics.charite.de/rhythm/ 394-416 429-459 481-499 506-528 533-555 562-581 631-651

16 SACS MEMSAT2 www.sacs.ucsf.edu/cgi-bin/memsat.py 483-499 506-529 536-556 630-646

17 OCTOPUS octopus.cbr.su.se/ 394-414 428-448 477-497 506-526 536-556 626-646

18 SPOCTOPUS octopus.cbr.su.se/ 394-404 427-447 466-496 506-526 535-555 627-647

19 SCAMPI-seq scampi.cbr.su.se/ 394-414 479-499 505-525 531-551 553-573 626-646

20 SCAMPI-msa topcons.cbr.su.se/ 394-414 428-448 477-497 506-526 536-556 626-646

21 PRODIV topcons.cbr.su.se/ 393-413 429-449 470-490 506-526 536-556 631-651

22 PRO opcons.cbr.su.se/ 393-413 429-449 470-490 506-526 536-556 630-650

23 TOPCONS topcons.cbr.su.se/ 394-414 429-449 477-497 506-526 536-556 626-646

24 TOPCONS-single single.topcons.net/ 394-414 479-499 507-527 536-556 630-650

25 MINNOU minnou.cchmc.org/ 373-387 393-413 421-458 466-495 504-530 536-553 562-582

26 TopPred 0.01 mobyle.pasteur.fr 396-416 427-447 481-501 505-525 536-556 630-650

27 EMBOSS-tmap mobyle.pasteur.fr/data/jobs/tmap 388-416 483-503 515-535 547-567 626-645

28 MEMSAT-SVM-tim bioinf.cs.ucl.ac.uk/web_servers/ 397-414 427-447 467-497 506-530 534-554 568-583 629-650

29 MEMSAT-tim bioinf.cs.ucl.ac.uk/web_servers/ 391-415 475-499 516-540 624-648

**Supplementary Table 2 Secondary helical structure prediction of human ABCG2.**

The table indicates the algorithms and online sources used for TMH prediction. The residues predicted to be within a given helical structures are presented in the table.

| **Programs** | **Sources** | **Amino acid residues** | | | | | | |
| --- | --- | --- | --- | --- | --- | --- | --- | --- |
| Jpred (Jnet)-H region | <http://www.compbio.dundee.ac.uk/www-jpred/> | 85-94 | 136-142 | 154-168 | 177-179 | 191-201 | 218-234 | 246-253 |
|  |  | 270-278 | 291-298 | 315-317 | 324-337 | 340-354 | 373-389 | 392-413 |
|  |  | 420-450 | 453-459 | 467-496 | 503-531 | 535-551 | 563-569 | 573-584 |
|  |  | 610-616 | 626-652 |  |  |  |  |  |
| MINNOU | <http://minnou.cchmc.org/> | 86-95 | 134-146 | 154-168 | 171-174 | 191-201 | 218-233 | 246-253 |
|  |  | 270-279 | 291-299 | 326-337 | 341-352 | 373-388 | 392-414 | 420-460 |
|  |  | 466-496 | 503-530 | 534-551 | 562-586 | 611-617 | 625-651 |  |
| 1D-PSIPRED | [http://biomine.ece.ualberta.ca](http://biomine.ece.ualberta.ca/) | 27-30 | 61-64 | 86-94 | 114-119 | 136-149 | 154-168 | 171-175 |
|  |  | 178-185 | 188-201 | 218-234 | 246-252 | 255-260 | 270-279 | 285-287 |
|  |  | 291-298 | 310-312 | 315-318 | 322-336 | 339-352 | 372-389 | 392-413 |
|  |  | 419-460 | 466-495 | 503-551 | 564-587 | 610-617 | 625-651 |  |
| PSIPRED-Tim | <http://bioinf.cs.ucl.ac.uk/psipred/> | 86-94 | 136-145 | 154-167 | 191-202 | 218-233 | 248-253 | 272-279 |
|  |  | 290-298 | 310-315 | 326-337 | 340-352 | 373-389 | 392-413 | 420-439 |
|  |  | 444-458 | 466-495 | 503-530 | 533-552 | 562-571 | 573-584 | 610-616 |
|  |  | 626-650 |  |  |  |  |  |  |
| EMBOSS-garnier | <http://mobyle.pasteur.fr/data/jobs/garnier/> | 24-39 | 59-66 | 91-96 | 136-154 | 157-162 | 168-176 | 221-231 |
|  |  | 254-262 | 270-274 | 276-278 | 292 | 305-321 | 327-338 | 342-363 |
|  |  | 444-457 | 475-478 | 480-482 | 496-497 | 501-531 | 537-546 | 549-552 |
|  |  | 610-616 | 630-637 | 640-652 |  |  |  |  |
| PREDATOR 2.1.2 | <http://mobyle.pasteur.fr/data/jobs/predator/> | 25-32 | 40-44 | 57-66 | 87-96 | 140-153 | 158-168 | 219-233 |
|  |  | 252-261 | 303-318 | 327-336 | 342-351 | 374-382 | 394-398 | 403-412 |
|  |  | 446-453 | 473-477 | 485-498 | 503-529 | 536-552 | 559-569 | 629-652 |

**Supplementary Table 3 Oligonucleotide primers used to generate ABCG2 mutations.**

| **Mutants** | **Forward primer (5'->3')** | **Reverse primer (5'->3')** |
| --- | --- | --- |
| K452E | CTTTGTGGTAGAGgagAAGCTCTTCATAC | GTATGAAGAGCTTctcCTCTACCACAAAG |
| K453E | GTGGTAGAGAAGgagCTCTTCATACATG | CATGTATGAAGAGctcCTTCTCTACCAC |
| R465E | GCGGATACTACgaaGTGTCATCTTATTTC | GAAATAAGATGACACttcGTAGTATCCGC |
| K473E | CTTATTTCCTTGGAgaaCTGTTATCTG | CAGATAACAGttcTCCAAGGAAATAAG |
| K647E | CAATTGCCTACCTGgaaTTGTTATTTCTTAA | TTAAGAAATAACAAttcCAGGTAGGCAATTG |
| K652E | AATTGTTATTTCTTgaaAAATATTCTTAAAT | ATTTAAGAATATTTttcAAGAAATAACAATT |
| K653E | GTTATTTCTTAAAgaaTATTCTTAAATTG | CAATTTAAGAATAttcTTTAAGAAATAAC |
| E446K | GTTTCAGCCGTGaAACTCTTTGTG | CACAAAGAGTTtCACGGCTGAAAC |
| E446A | GTTTCAGCCGTGGcACTCTTTGTG | CACAAAGAGTgCCACGGCTGAAAC |
| E446D | GTTTCAGCCGTGGAtCTCTTTGTG | CACAAAGAGaTCCACGGCTGAAAC |
| E451R | CTCTTTGTGGTAaggAAGAAGCTCTTC | GAAGAGCTTCTTcctTACCACAAAGAG |
| E451A | CTCTTTGTGGTAgcgAAGAAGCTCTTC | GAAGAGCTTCTTcgcTACCACAAAGAG |
| E451D | CTCTTTGTGGTAgacAAGAAGCTCTTC | GAAGAGCTTCTTgtcTACCACAAAGAG |
| E458R | GCTCTTCATACATagaTACATCAGCGGATAC | GTATCCGCTGATGTAtctATGTATGAAGAGC |
| E458A | GCTCTTCATACATgcaTACATCAGCGGATAC | GTATCCGCTGATGTAtgcATGTATGAAGAGC |
| E458D | GCTCTTCATACATgacTACATCAGCGGATAC | GTATCCGCTGATGTAgtcATGTATGAAGAGC |
| D477R | GAAAACTGTTATCTagaTTATTACCAATG | CATTGGTAATAAtctAGATAACAGTTTTC |
| D477A | GAAAACTGTTATCTgcaTTATTACCAATG | CATTGGTAATAAtgcAGATAACAGTTTTC |
| D477E | GAAAACTGTTATCTgaaTTATTACCAATG | CATTGGTAATAAttcAGATAACAGTTTTC |
| K86A | CAGGTGGAGGCgcATCTTCGTTATTAG | CTAATAACGAAGATgcGCCTCCACCTG |
| K86M | CAGGTGGAGGCAtgTCTTCGTTATTAG | CTAATAACGAAGAcaTGCCTCCACCTG |
| R137E | CACTCTGACGGTGgaaGAAAACTTACAG | CTGTAAGTTTTCttcCACCGTCAGAGTG |
| R137A | CACTCTGACGGTGgcaGAAAACTTACAG | CTGTAAGTTTTCtgcCACCGTCAGAGTG |
| R137K | CACTCTGACGGTGaaaGAAAACTTACAG | CTGTAAGTTTTCtttCACCGTCAGAGTG |
| E138R | CTGACGGTGAGAagaAACTTACAGTTC | GAACTGTAAGTTTCTTCTCACCGTCAG |
| E138K | CTGACGGTGAGAaaaAACTTACAGTTC | GAACTGTAAGTTtttTCTCACCGTCAG |
| E138H | CTGACGGTGAGAcacAACTTACAGTTC | GAACTGTAAGTTgtgTCTCACCGTCAG |
| E138A | CTGACGGTGAGAgcaAACTTACAGTTC | GAACTGTAAGTTtgcTCTCACCGTCAG |
| E138D | CTGACGGTGAGAgacAACTTACAGTTC | GAACTGTAAGTTgtcTCTCACCGTCAG |
| D171R | GAGTTAGGTCTGagaAAAGTGGCAGAC | GTCTGCCACTTTtctCAGACCTAACTC |
| K172E | GTTAGGTCTGGATgaaGTGGCAGACTCC | GGAGTCTGCCACttcATCCAGACCTAAC |
| H375A | CCTCCTTCTGTgcTCAACTCAGATG | CATCTGAGTTGAgcACAGAAGGAGG |
| Q376E | CCTTCTGTCATgAACTCAGATGGG | CCCATCTGAGTTcATGACAGAAGG |
| R378E | CTGTCATCAACTCgaATGGGTTTCTAAG | CTTAGAAACCCATtcGAGTTGATGACAG |
| W379A | CATCAACTCAGAgcGGTTTCTAAGC | GCTTAGAAACCgcTCTGAGTTGATG |
| K382E | CAGATGGGTTTCTgAGCGTTCATTC | GAATGAACGCTcAGAAACCCATCTG |
| K382R | CAGATGGGTTTCTAgGCGTTCATTC | GAATGAACGCcTAGAAACCCATCTG |
| R383E | GGGTTTCTAAGgagTCATTCAAAAAC | GTTTTTGAATGActcCTTAGAAACCC |
| R383K | GGTTTCTAAGaagTCATTCAAAAAC | GTTTTTGAATGActtCTTAGAAACC |
| K386E | GCGTTCATTCgAAAACTTGCTGGG | CCCAGCAAGTTTTcGAATGAACGC |
| N387E | CGTTCATTCAAAgAgTTGCTGGGTAATC | GATTACCCAGCAAcTcTTTGAATGAACG |
